# Precision Synthesis of Conjugated Polymer Films by Surface-Confined Stepwise Sonogashira Cross-Coupling

**DOI:** 10.3390/molecules29225466

**Published:** 2024-11-20

**Authors:** Sang Gil Youm, Mitchell T. Howell, Chien-Hung Chiang, Lu Lu, Neepa M. K. Kuruppu Arachchige, John F. Ankner, Joseph Strzalka, Yaroslav Losovyj, Jayne C. Garno, Evgueni E. Nesterov

**Affiliations:** 1 Department of Chemistry and Biochemistry, Northern Illinois University, DeKalb, IL 60115, USA; 2 Department of Chemistry, Louisiana State University, Baton Rouge, LA 70803, USA; 3 Spallation Neutron Source, Oak Ridge National Laboratory, Oak Ridge, TN 37831, USA; 4 X-Ray Science Division, Argonne National Laboratory, Argonne, IL 60439, USA; 5 Department of Chemistry, Indiana University, Bloomington, IN 47401, USA; ylozovyy@iu.edu

**Keywords:** Sonogashira cross-coupling, stepwise polymerization, polymer thin films, fluorescent sensor, excitation energy transfer, detection of nitroaromatic compounds

## Abstract

Thin films of poly(arylene ethynylene)-conjugated polymers, including low-energy-gap donor–acceptor polymers, can be prepared via stepwise polymerization utilizing surface-confined Sonogashira cross-coupling. This robust and efficient polymerization protocol yields conjugated polymers with a precise molecular structure and with nanometer-level control of the organization and the uniform alignment of the macromolecular chains in the densely packed film. In addition to high stability and predictable and well-defined molecular organization and morphology, the surface-confined conjugated polymer chains experience significant interchain electronic interactions, resulting in dominating intermolecular π-electron delocalization which is primarily responsible for the electronic and spectroscopic properties of polymer films. The fluorescent films demonstrate remarkable performance in chemosensing applications, showing a turn-off fluorescent response on the sub-ppt (part per trillion) level of nitroaromatic explosives in water. This unique sensitivity is likely related to the enhanced exciton mobility in the uniformly aligned and structurally monodisperse polymer films.

## 1. Introduction

The performance of electronic, optoelectronic, and sensing devices based on thin films of conjugated polymers (CPs) depends on both the polymers’ intrinsic electronic properties and on the nanoscale and mesoscale organization of the macromolecules in bulk materials [1,2,3,4,5,6]. The electronic properties of conjugated polymers depend on the polymer molecular structure, and can be effectively adjusted via chemical synthesis. However, even the best synthetic methods (such as those relying on controlled, or “living” polymerization protocols [7,8]) deliver polymers with a distribution of molecular weights (polydispersity > 1.0), and do not allow “precision” synthesis. From the morphology control standpoint, the traditional methods for the fabrication of thin films that rely on the solution processing (e.g., spin casting) of pre-synthesized CPs do not provide sufficient control over molecular organization, macromolecular alignment, and the morphology of the resulting films. Recently developed surface-initiated catalyst–transfer polymerization enables better control over the alignment and nanoscale morphology of CP thin films, but it is seriously limited to only a few classes of CPs of relatively simple structures (such as polythiophenes and poly(*p*-phenylene)s) [9,10,11,12,13,14], and it does not allow the precision synthesis of CPs.

**Figure 1:**
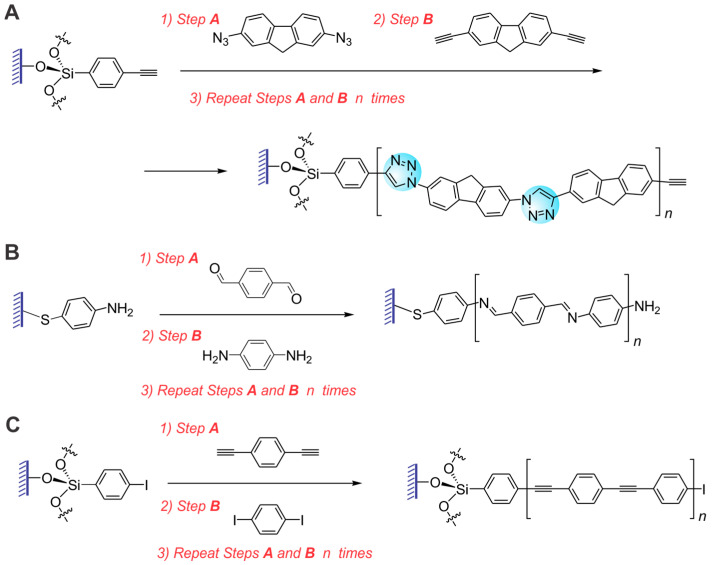
Preparation of conjugated polymer thin films by surface-confined stepwise precision polymerization through (**A**) Cu-catalyzed azide–alkyne click reaction; (**B**) formation of polyimine via reaction of aromatic bis-aldehyde and bis-amine; (**C**) surface-confined Sonogashira cross-coupling *(this work)*.

Instead, we decided to focus on stepwise surface-confined polymerization—a promising but rarely used approach to preparing polymer thin films with precise molecular structure and nanometer-level control of the molecular organization and alignment. This method (sometimes referred to as molecular layer deposition [15]) has been developed for the functionalization of various surfaces with thin layers of insulating organic or metal–organic polymers [16,17,18,19]. In this process, surface-immobilized polymer brushes are prepared through a series of repetitive, one-per-step, additions of monomers to the growing end of a polymer chain. Therefore, obtaining polymers with precise molecular compositions and uniform molecular organizations would be possible with a proper selection of monomer compounds. Although stepwise polymerization has been explored for various insulating polymer films, it has not been well studied in the preparation of conjugated polymer thin films. For the stepwise polymerization to be successful, the chemical reactions used for the monomer coupling have to be highly efficient (i.e., provide nearly quantitative, close to 100% conversion in a relatively short reaction time) which limits the range of the coupling reactions that can be explored. Among such reactions, the Cu-catalyzed azide–alkyne click (CuAAC) reaction has been previously used in surface-confined stepwise polymerization (Figure 1A) [20,21,22]. Alternating the exposure of the alkyne-functionalized substrate to the solutions of bis-azide and bis-alkyne monomers enabled the preparation of polymer thin films with up to a few nanometers of thickness. However, the 1,2,3-triazole units formed in the CuAAC reaction limit the π-electron delocalization along the macromolecular chain, resulting in the polymers showing somewhat less “conjugated” characteristics [23]. Alternatively, Frisbie et al. have developed a surface-confined stepwise polymerization approach toward poly(*p*-phenylene imine) thin films via the reliable and efficient reaction of aromatic bis-aldehyde and bis-amine monomers (Figure 1B) [24]. Aside from these two exotic classes of CPs, preparation of other, more conventional classes of CPs via surface-confined precision polymerization has not been demonstrated, and the applicability of the corresponding coupling reactions for this process has not been evaluated. Poly(*p*-phenylene ethynylene)s (PPEs) of various structures represent a popular class of CPs for fluorescent chemosensing applications [25,26]. In this work, we describe the successful and efficient preparation of thin films of various PPEs via stepwise surface-confined precision polymerization utilizing Sonogashira cross-coupling (Figure 1C). We demonstrated the preparation of structurally precise PPE thin films with nanometer-level control of the molecular organization and alignment. We have also studied, for the first time, the electronic and spectroscopic properties of the resulting thin films. In addition, we demonstrated how the precise control of the structure and morphology in the CP thin films can dramatically improve their fluorescent chemosensing performance, resulting in sub-ppt (part-per-trillion) detection sensitivity towards the nitroaromatic explosive compounds.

## 2. Results and Discussion

### 2.1. Surface-Confined Polymerization via Stepwise Sonogashira Cross-Coupling

**Figure 2:**
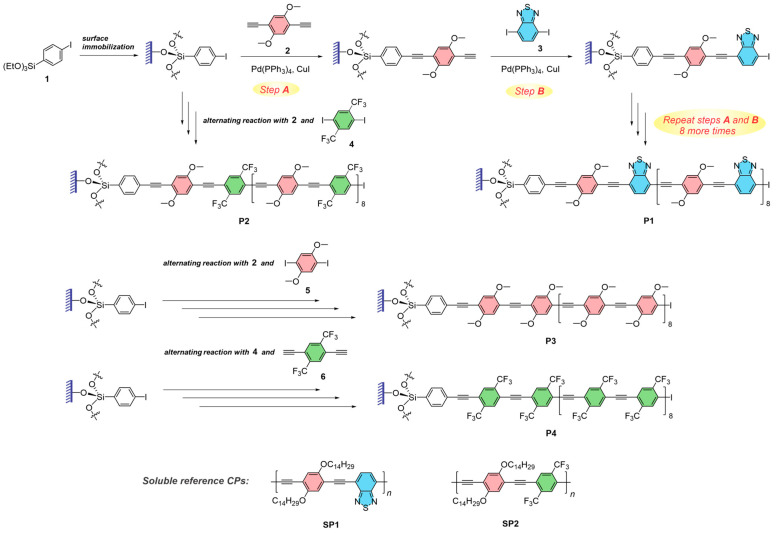
Preparation of poly(arylene ethynylene) thin films by surface-confined stepwise precision polymerization using Sonogashira cross-coupling, and chemical structures of the surface-confined CPs **P1**–**P4**, as well as soluble reference polymers **SP1** and **SP2**.

**Figure 3:**
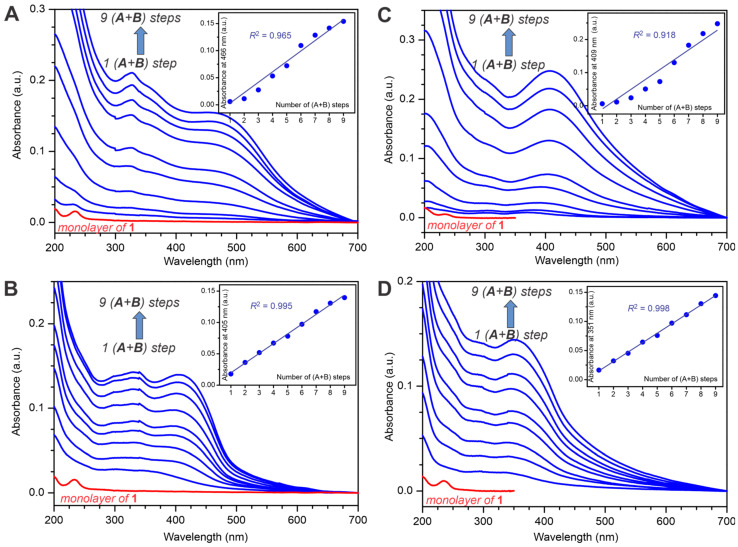
UV/vis spectroscopy monitoring of the evolution of thin films of **P1** (**A**), **P2** (**B**), **P3** (**C**), and **P4** (**D**). Red traces correspond to the monolayer of precursor **1**. Inserts show the absorbance increase as a function of the number of coupling steps, and straight lines correspond to the linear least squares fits to the datasets.

In the first step of surface-confined polymerization, we prepared covalently immobilized monolayers of 4-(triethoxysilyl)-1-iodobenzene **1** on pre-activated quartz slides (Figure 2). The formation of the monolayer of **1** was confirmed via UV/vis absorption spectroscopy based on the appearance of a characteristic absorption band at 240 nm (Figure 3). Prior to carrying out full-fledge stepwise polymerization, it was essential to quantify the reaction conversion efficiency in the surface-confined Sonogashira coupling protocol. We relied on X-ray photoelectron spectroscopy (XPS) for this study as XPS is highly sensitive for evaluating the composition of monolayers and thin films. We chose to use iodine signals since iodine is not present in the product of the Sonogashira cross-coupling of **1** with bis-acetylene monomer **2** (Figure 2), and the percent conversion in the coupling reaction could be estimated based on the atomic ratio of the residual iodine to the carbon (derived from the intensities of the corresponding XPS signals; see Supporting Information for details on the analysis). Thus, even for a 30 min reaction time, the conversion was at least 92%, and it increased to at least 96% and >99% for 1 h and 2 h reaction times, respectively. Therefore, with this experiment, we confirmed that surface-confined Sonogashira cross-coupling was indeed an efficient and robust reaction suitable for precision stepwise polymerization.

The preparation of thin films of various PPE polymers **P1**–**P4** via surface-confined polymerization was carried out through the alternating immersion of a slide modified with a monolayer of iodo-precursor **1** in the solutions of corresponding bis-iodo and bis-acetylene monomers (e.g., monomers **2** and **3** in the case of polymer **P1**) also containing catalytic amounts of Pd(PPh_3_)_4_ and CuI. First, the monolayer-modified slide reacted with a solution of the bis-acetylene monomer **2** at 40 °C for 1 h to yield an alkyne-terminated film (step A, Figure 2). After it was thoroughly rinsed with toluene, the alkyne-terminated thin film was immersed in a solution of bis-iodo monomer **3** at 40 °C for 1 h, followed by ultrasonication in toluene to yield a surface-confined iodo-terminated film (step B, Figure 2). The ultrasonication step was essential to ensure that any reaction products not chemically bound to the quartz slide (e.g., any solution-formed coupling byproducts) would be completely removed from the substrate and therefore would not become part of the produced thin films. The sequence of the two alternating Sonogashira coupling steps A and B was repeated 8 more times to yield a thin film of the monodisperse conjugated polymer **P1**.

Similarly to the polymer film **P1**, we used stepwise polymerization based on Sonogashira cross-coupling to prepare thin films of the CPs **P2**–**P4** (Figure 2). In all the cases, the stepwise polymerization occurred smoothly and efficiently, and generated almost no polymer byproducts in the monomer solutions. The efficiency of the surface-confined Sonogashira cross-coupling remained high throughout each step, as was checked using XPS (Figure S3 in Supporting Information). To confirm the monodisperse nature of the resulting macromolecules, we also carried out stepwise preparation of the polymer **P3** confined to the surface of 300 nm diameter silica microspheres. Following the preparation, the silica core was dissolved in hydrofluoric acid and the resulting oligomers were analyzed via gel-permeation chromatography (GPC) to determine the dispersity index. Due to the absence of the solubilizing groups, only the oligomer obtained via 2 sequential (A + B) steps (**P3-2**) was found to be sufficiently soluble in common solvents; the oligomer obtained after 4 (A + B) steps (**P3-4**) was barely soluble to allow GPC analysis, and the materials obtained after six ot more (A + B) steps were found completely insoluble and could not be analyzed. Nevertheless, both **P3-2** and **P3-4** showed a dispersity index *Ð* < 1.03 (Figure S4 in Supporting Information), indicating the high efficiency of the surface-confined Sonogashira cross-coupling and the monodisperse nature of the prepared oligomer/polymer chains.

The evolution of the thin films of the polymers **P1**–**P4** was monitored using UV/vis absorption spectroscopy, which exhibited an approximately linear intensity increase in the corresponding CP absorption bands (Figure 3). The thin films of polymers **P1** and **P3** demonstrated some wider deviation from the linear behavior (Figure 3A,C, inserts). This could be related to the intermolecular electronic interactions between the oligomer chains or simply to an experimental error of the absorption measurements; however, we did not further investigate such deviation from linearity. Remarkably, all the surface-confined polymer films demonstrated excellent stability, owing to the covalent bonding between the macromolecules and the substrate surface, and no film deterioration was noticed even upon ultrasonication in common organic solvents (such as chloroform, toluene, etc.) as was evidenced by the virtually unchanged UV/vis absorption spectra after ultrasonication.

In principle, the precision polymerization process could be carried out to more than nine (A + B) repeating steps. For example, we accomplished the preparation of a **P3** thin film utilizing a sequence of 20 (A + B) steps (see Figure S5 in Supporting Information for the UV/vis absorption spectrum). However, performing so many repeating steps is time consuming and practically unfeasible. Furthermore, aiming at chemosensing applications, the spectroscopic and electrochemical characteristics of the thin films were found to be virtually invariable with respect to the number of coupling steps (vide infra). Thus, a sequence of nine (A + B) steps was chosen as a reasonable practical compromise.

### 2.2. Spectroscopic and Electrochemical Properties of Surface-Confined Polymers

**Figure 4:**
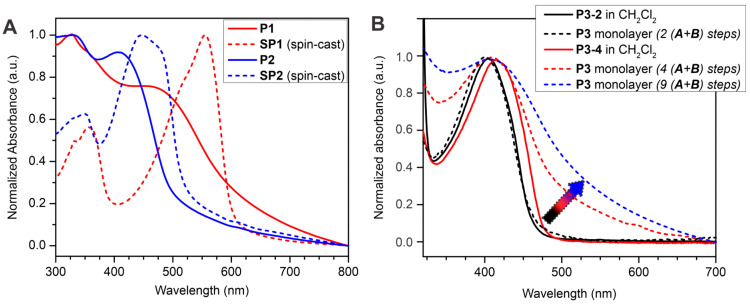
(**A**) Comparison of UV/vis absorption spectra of surface-confined thin films **P1** and **P2** with the spectra of spin-cast thin films of the soluble analogs **SP1** and **SP2**. (**B**) Evolution of the UV/vis absorption spectra of the surface-confined film **P3** and its comparison with the solution spectra of the oligomers **P3-2** and **P3-4**. The arrow indicates the evolution of the low-energy tail of the absorption band.

Whereas the surface-confined CP films **P1**–**P4** exhibited a predictable increase in the UV/vis absorption intensity as a function of the number of added repeating units, the wavelengths corresponding to the absorption maxima remained nearly constant after the second (A + B) coupling cycle and subsequent cycles. To elucidate this unusual spectroscopic trend, we prepared the polymers **SP1** and **SP2** as soluble analogs of the polymers **P1** and **P2** (Figure 2) using a conventional solution step-growth polymerization protocol. A comparison of the UV/vis absorption spectra of the spin-cast polymer films revealed that the polymers **SP1** and **SP2** displayed bathochromically shifted maxima of the absorption bands relative to the spectra of the structurally similar surface-confined polymers **P1** and **P2** (Figure 4A). At the same time, the surface-confined **P1** and **P2** polymer films exhibited broader absorption bands, with intense low-energy tails at the long-wavelength onsets extending beyond those of the conventionally prepared polymers **SP1** and **SP2**. In a further experiment, we compared absorption spectra of the soluble oligomers **P3-2** and **P3-4** in dilute solutions with the spectra of the same compounds in surface-confined monolayers (Figure 4B). Although the wavelengths of the absorption maxima were not significantly different (and were also similar to the absorption maximum of the **P3** thin film prepared using the nine (A + B) coupling steps), the surface-confined monolayers showed a small hypsochromic shift (4 to 5 nm) relative to the corresponding spectra in the solution. Furthermore, the intensity of the long-wavelength tail in the spectra of the surface-confined monolayers markedly and consistently increased with the increasing oligomer length (marked with an arrow in Figure 4B). Such spectroscopic behavior could be associated with the dominating presence of interchain H-aggregates, resulting in the prevalent electronic excitation delocalized between the π-conjugated chains [27]. The formation of H-aggregates would require symmetrical uniform alignment of surface-confined chains stacked in a coplanar face-to-face fashion. A similar hypsochromic shift was observed by Collard et al. in the absorption spectra of closely stacked short oligo(*p*-phenylene ethynylenes); there was also a characteristic low-energy tail in the absorption spectra that was not present in the unstacked oligomers and was likely originating from the intermolecular coupling of the π-electron systems [28]. Thus, we can assume that surface-confined π-conjugated chains of the CPs **P1**–**P4** experienced significant interchain electronic interactions, resulting in dominating intermolecular π-electron delocalization which was chiefly responsible for the electronic and spectroscopic properties of the surface-confined π-conjugated molecules, even at a low number of the coupling steps. Such enhanced intermolecular electronic delocalization would necessarily facilitate exciton migration within the films, thus making them even more suitable for fluorescent sensing applications.

To further understand the electronic properties of the surface-confined CP **P1**–**P4** thin films and their suitability for chemosensing applications, we carried out cyclic voltammetry (CV) studies (for this purpose, the polymer films were prepared on glass substrates coated with indium tin oxide (ITO) following the procedure described above for the quartz substrates). The films displayed sharp irreversible oxidation and/or reduction peaks consistent with the monodisperse nature of the surface-confined polymers (Figure S6 in Supporting Information). The position of the onsets of the CV peaks allowed the estimation of the polymers’ frontier molecular orbital (HOMO and LUMO) levels, as well as electronic energy gaps (Table 1). The electrochemical energy gap values (*E*_g_^ec^) were in good agreement with the values obtained from the long-wavelength onset of the UV/vis absorption spectra (*E*_g_^opt^). As expected, the energy of HOMO and LUMO could be effectively tuned using differently substituted monomeric units, with, for example, thin films of the donor–acceptor polymer **P1** displaying the lowest HOMO-LUMO energy gap (*E*_g_^ec^ 1.40 eV) as compared to the other polymer films. Thus, the structural alteration of surface-confined CP molecules is an efficient way to adjust the electronic properties of the resulting thin films to suit specific applications. We also investigated the dependence of the electrochemical characteristics of the CP films on the number of the coupling steps (using polymer **P2** as an example). In corroboration with the trend of the invariance of UV/vis absorption maxima, thin films of the polymer **P2** showed practically no change in the position of the electrochemical oxidation potential upon the increasing number of (A + B) coupling steps; it also showed a near-linear increase in the oxidation current according to the number of the coupling steps (Figure S7 in Supporting Information).

### 2.3. Morphology Studies and Chain Alignment in the Surface-Confined Films

**Figure 5:**
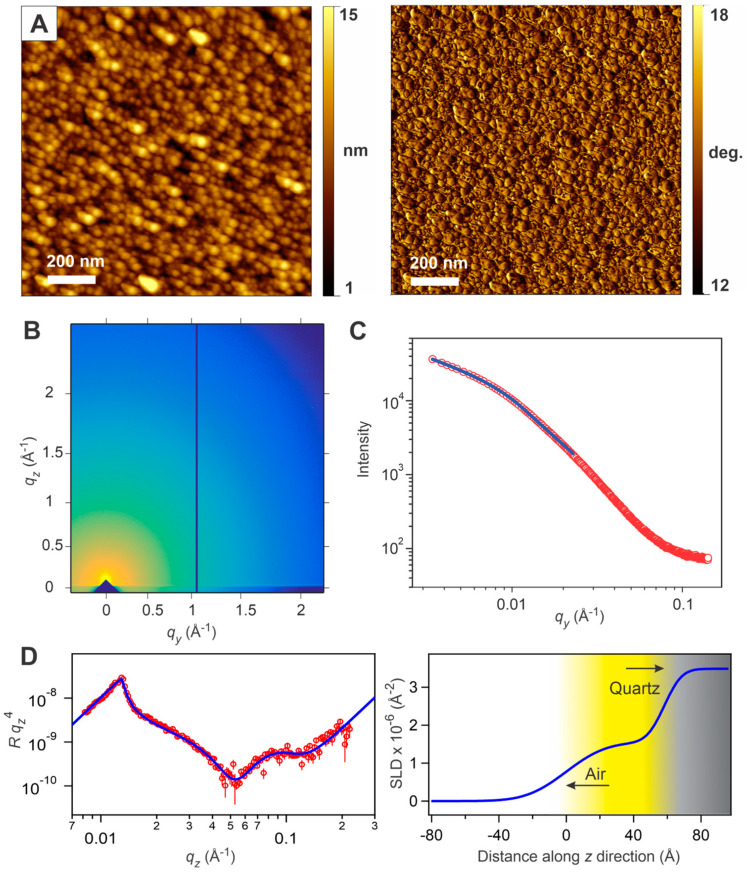
Structural and morphological characterization of a **P1** thin film prepared by surface-confined polymerization on a quartz substrate: (**A**) morphology of the film as viewed with contact mode AFM—topography (left image) and a simultaneously acquired lateral force image (right image). (**B**) Two-dimensional GIWAXS image. (**C**) GISAXS horizontal line trace for the film in (**B**) (red circles) and fitting these data using a modified Guinier–Porod model (blue trace). (**D**) Neutron reflectometry study of a partially deuterated **P1** thin film prepared using 7 (A + B) steps—reflectivity data for the film (red circles) and best fit to the data (blue solid line) (left plot), and scattering length density (SLD) profile based on the best-fit model (right plot).

Both the spectroscopic and electrochemical findings indicated a high degree of interchain π-electron interactions, potentially stemming from the unique structural and morphological uniformity of the surface-confined precision CPs, and prompted a more detailed investigation of the surface-confined film structure and morphology, using the CPs **P1** and **P2** as representative samples. The surface morphology of the thin films was characterized using atomic force microscopy (AFM). The thin films exhibited the uniform surface coverage of closely packed circular domains with an average diameter ranging from 35 nm to 60 nm (Figure 5A and Figure S9 in Supporting Information). The lateral force images sensitively provided information on the chemical homogeneity of the samples at the molecular level, and revealed that the chemical composition of the samples was quite homogeneous and uniform throughout the areas that were sampled. The observed surface domains did not result from the inhomogeneity of the quartz substrate, as the polished quartz slides used in this study showed a nearly featureless and flat surface (Figure S10 in Supporting Information). The formation of circular domains has been previously observed for the polymer films prepared via surface-confined CuAAC polymerization [22], as well as in the case of the polythiophene thin films prepared via surface-initiated Kumada catalyst–transfer polymerization [13]. The uniform surface morphology featuring the circular domains was highly reproducible throughout this study, and appeared as a nanoscale hallmark of the surface-confined polymerization.

Grazing incidence wide- and small-angle X-ray scattering (GIWAXS and GISAXS, respectively) are common techniques used to evaluate the structure and morphology of polymer thin films [30]. GIWAXS studies of the surface-confined thin films revealed no distinct diffraction features (Figure 5B and Figure S11 in Supporting Information), indicating that these polymer films were essentially amorphous, with no crystalline chain packing. Although the formation of the interchain H-aggregates (postulated based on UV/vis absorption spectral features, vide supra) would assume some kind of close packing of the surface-confined polymer chains, such packing did not result in crystalline polymer film. The lack of crystallinity could originate from the large tilting angle of the surface-immobilized polymer chains, precluding their crystallization. Indeed, such tilt was found in the optical anisotropy experiments described below. The horizontal linecuts of the GISAXS data featured a “Guinier knee” due to the predominance of lateral nanoscale domains within the films (Figure 5C and Figure S11 in Supporting Information). Fitting these data using a combined Porod plus Guinier model [31] allowed us to estimate the size of the lateral domains being within 10 to 12 nm. These values were significantly smaller than the diameters of the surface circular domains observed in the AFM studies (vide supra). These uniform smaller-size domains found in the GISAXS experiments could be attributed to the primary nanoscale scattering objects of the surface-confined polymer chains that represent the morphological building blocks of the bulk surface-confined films.

The important information about the cross-sectional characteristics of the films could be obtained using neutron reflectometry (NR), which is a powerful tool to study organic thin films, due to its ability to enhance the contrast of specific components of the films using site-selective deuterium labeling [32,33,34]. Partially deuterated **P1** thin films for NR studies were prepared using a 1,4-bis(deuteromethoxy)-2,5-bis(ethynyl)benzene (**2-D_6_**) monomer (for practical considerations, the films for this study were prepared using seven (A + B) coupling steps). The NR data were acquired upon reflecting a neutron beam from the polymer/air interface, and produced a reflectivity pattern, which was modeled using a one-layer slab model fitting approach to achieve the best fit of the experimental data (Figure 5D). The scattering length density (SLD) profile obtained based on this fitting model indicated a total film thickness of 5.8 nm. This value was substantially different from the calculated length of the rod-like **P1** molecule prepared in seven (A + B) steps (10.3 nm based on a semi-empirical AM1 geometry optimization), and indicated some significant tilt of the surface-confined molecules toward the substrate. The constant SLD value across the thin film (1.55 × 10^−6^ Å^−2^) corresponded to the mass density of the film of 0.47 g cm^−3^. This density was lower than the previously reported density of the crystalline samples of (*p*-phenylene ethynylene) short oligomers (which ranges from 1.0 to 1.2 g cm^−3^) [35,36], and, when considered together with the broad diffuse polymer/air interface featured on the SLD profile, was consistent with the observation of distinct circular domains (rather than uniform featureless film) in the AFM imaging experiments.

**Figure 6:**
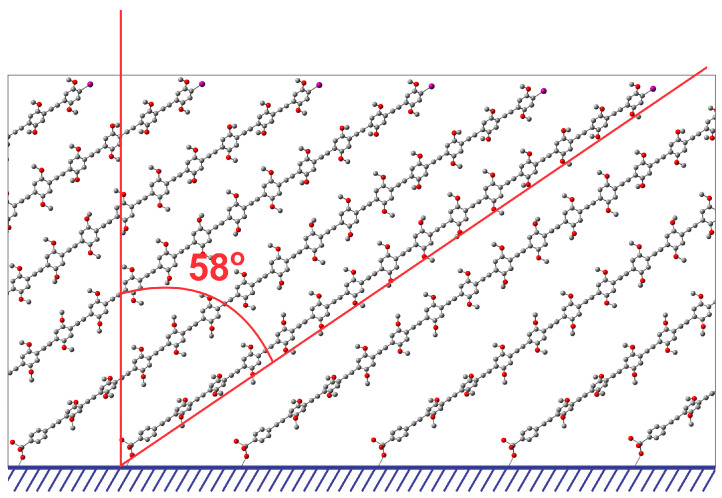
Definition of the tilting angle *ψ* (58°) as determined by polarized absorption spectroscopy.

Optical absorption anisotropy of **P3** thin films could be used to assess the preferential alignment of the surface-confined conjugated chains, and it was studied using polarized absorption spectroscopy. The linear relationship between the dichroic ratio *D*_HV_ and sin^2^*δ* (where *δ* is the thin-film sample twisting angle—the angle between the incident light direction and the normal to the polymer film) indicated the highly anisotropic nature of the film with uniform alignment of the rod-like surface-confined chains [37]. From the slope of this relationship, we calculated the tilting angle *ψ* of 58° between the long molecular axis of the surface-confined chains and the normal to the substrate surface (Figure 6). Based on this value of the tilting angle *ψ* and assuming that it was of a similar value for the polymer **P1** thin films, the film thickness of the polymer **P1** used for the NR experiments was calculated at 5.5 nm, which was indeed remarkably close to the value obtained from the SLD profile (5.8 nm). From the value of *ψ*, the size of the projection of the polymer **P1** chain on the plane of the substrate surface was estimated at 11.1 nm, and it was remarkably close to the size of the lateral scattering objects derived in the GISAXS experiments (10–12 nm, vide supra), indicating that the chemically monodisperse aligned **P3** molecules were indeed the primary X-ray scattering objects. The consistency between the results obtained using different experimental techniques indicated the high structural uniformity and molecular precision of the surface-confined CP thin films, where the significantly tilted monodisperse CP chains were packed side-by-side in near-parallel to the substrate surface fashion, forming nanoscale domains within the relatively thin films. Such structural arrangement could facilitate both the formation of interchain H-aggregates between the short interacting segments of the CP chains, and related through-space excitation delocalization in the films. This organization could also provide a rapid exciton expansion to the film surface where the binding of nitroaromatic analytes occurs [38].

### 2.4. Fluorescent Chemosensing Studies with Polymer P3 Thin Films

In order to demonstrate how the structural and morphological uniformity of the CP films prepared by precision stepwise polymerization can improve polymer film characteristics related to practical applications, we investigated the chemosensing performance of these materials. Conjugated polymers, and poly(arylene ethynylene)s in particular, provide a versatile platform for the design of amplifying fluorescent chemosensors [39]. Specifically, thin films of such polymers show superior detection sensitivity towards electron deficient analytes, such as nitroaromatic explosive compounds, which is related to the amplification of fluorescence quenching due to facile exciton transport within interacting π-electron conjugated systems [40,41]. Previously, we have demonstrated that the uniform orientation and molecular alignment of π-electron conjugated oligomers within a surface-immobilized monolayer resulted in the significant improvement of the chemosensing performance [42,43]. This improvement is primarily related to the increasing efficiency of intermolecular exciton migration originating from the parallel alignment of the conjugated oligomers’ transition dipole moments. Considering the intrinsic structural and morphological uniformity of the CP films prepared by precision stepwise synthesis, we expected that similar effects would be operational in this case as well. Therefore, we investigated the thin films of the electron-rich polymer **P3** for the fluorescent detection of 2,4-dinitrotoluene (2,4-DNT) in aqueous media, as this would be related to the field detection of the buried explosive devices in a real environment.

Since fluorescence quenching involves an electron transfer from the excited polymer to the LUMO of a quencher, the magnitude of the electron transfer Gibbs free energy change Δ*G*^0^ determines the thermodynamic “driving force” of the quenching process, and it was estimated using the Rehm–Weller equation [44]:

Δ*G*^0^ = *E*_LUMO_(Q) − *E*_HOMO_(**P3**) − *E*_00_ (1)

In this equation, *E*_HOMO_(**P3**), *E*_LUMO_(Q), and *E*_00_ are the HOMO energy of the polymer **P3** (from Table 1), the LUMO energy of a quencher (−3.5 eV for 2,4-DNT [45]), and the energy difference between the ground and first excited singlet states of the polymer **P3**, respectively. The value *E*_00_ (2.71 eV) was obtained from the intersecting wavelength of the normalized absorption and fluorescence spectra of the polymer **P3**. We found the electron transfer to be a highly exergonic process for 2,4-DNT, with a corresponding Δ*G*^0^ of −0.58 eV. As the polymer **P3** displayed the highest *E*_HOMO_ energy of the four studied polymers (Table 1), it would benefit from the highest exergonicity of the electron transfer process to improve chemosensing performance, and thus, it was selected for further fluorescent sensing studies.

**Figure 7:**
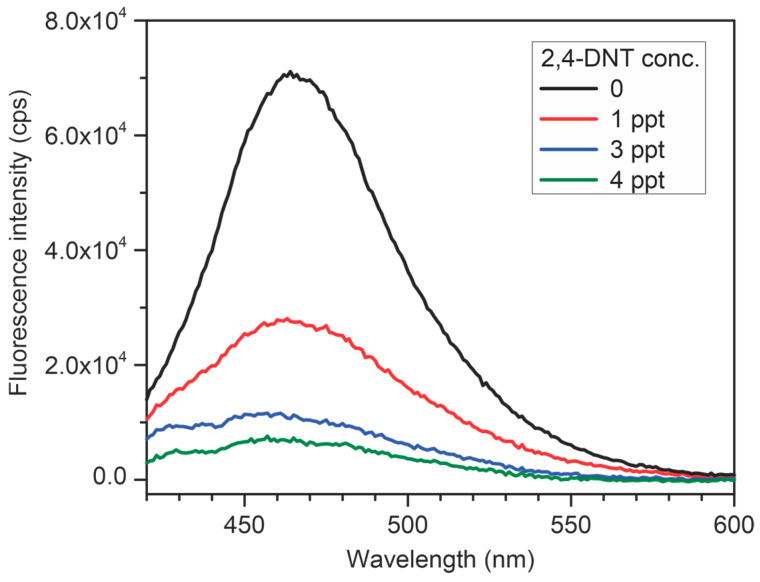
Quenching of fluorescence emission of a polymer **P3** thin film upon exposure to various concentration 2,4-DNT aqueous solutions.

Because the typical detection limit of fluorescent CP sensors for nitroaromatic compounds lies within the ppb (part-per-billion) range [46], we first immersed a **P3**-modified slide in an aqueous solution of 2,4-DNT with a 1 ppb concentration. We were surprised to find that fluorescence was completely quenched in that case. Indeed, the **P3** films showed a remarkable low-ppt (part-per-trillion) detection for 2,4-DNT (Figure 7). A comparable (albeit, still larger at 20 ppt) detection limit was previously achieved with a much more sophisticated enzyme-linked immunosorbent assay (ELISA) [47], but is not typical for fluorescent conjugated polymer sensors. Thus, this is an excellent example of enhancing useful practical properties in surface-confined precision CP films.

In addition to their remarkable fluorescent detection performance, the surface-confined polymer **P3** films demonstrated high stability (stemming from the covalent bonding of the polymer chains to the quartz substrate). This high stability made it possible to reset and reuse the **P3** polymer sensors. Specifically, washing the quenched **P3** films previously exposed to 2,4-DNT with ethanol resulted in the restoration of their fluorescent emission and allowed multiple-time use for the analyte detection without significant deterioration of the fluorescent properties (Figure S14 in Supporting Information). This possibility to reset and reuse the surface-confined CP fluorescent films could prove particularly useful in developing practical trace explosive detectors. Other applications (e.g., in light-emitting and other optoelectronic devices) could also benefit from these materials, and will be studied in due course.

## 3. Materials and Methods

### 3.1. General Procedures

All the reactions were performed under an atmosphere of dry nitrogen (unless mentioned otherwise). Column chromatography was performed on silica gel (Sorbent Technologies, Norcross, GA, USA, 60 Å, 40-63 μm) slurry packed into glass columns. Tetrahydrofuran (THF), toluene, and hexane were dried by passing through activated alumina, and *N*,*N*-dimethylformamide (DMF), by passing through activated molecular sieves, using a PS-400 Solvent Purification System from Innovative Technology, Inc. (Newburyport, MA, USA). The water content of the solvents was periodically controlled by Karl Fischer titration (using a DL32 coulometric titrator from Mettler Toledo, Columbus, OH, USA). Anhydrous USP grade ethanol for fluorescent detection studies was purchased from Decon Labs, Inc. (King of Prussia, PA, USA). Tetrabutylammonium hexafluorophosphate (Bu_4_NPF_6_) for electrochemical studies was acquired from Aldrich (St. Louis, MO, USA) and used after the additional recrystallization from ethanol. Isopropylmagnesium chloride (2.0 M solution in THF) was purchased from Acros Organic (Geel, Belgium), and was titrated with salicylaldehyde phenylhydrazone prior to use [48]. All the other reagents and solvents were obtained from Aldrich and Alfa Aesar (Haverhill, MA, USA) and used without further purification. The UV–visible spectra were recorded using an Agilent Cary 5000 UV-Vis-NIR spectrophotometer (Santa Clara, CA, USA). Fluorescence studies were carried out using a PTI QuantaMaster4/2006SE spectrofluorimeter (Photon Technology International, Edison, NJ, USA). ^1^H NMR spectra were recorded at 400 MHz and were reported in ppm downfield from tetramethylsilane. A GPC analysis of the polymers was performed with an Agilent 1100 chromatograph equipped with two PLgel 5 μm MIXED-C columns and one PLgel 5 μm 1000 Å column connected in a series, using THF as a mobile phase at a flow rate of 0.7 mL min^−1^, and calibrated against polystyrene standards. Indium tin oxide (ITO)-coated glass slides (25 × 75 × 1.1 mm polished float glass, 8-12 Ohm/sq. surface resistivity) were purchased from Delta Technologies, Ltd. (Loveland, CO, USA). Polished rectangular quartz slides (75 × 25 mm) were purchased from Chemglass (Vineland, NJ, USA). Electrochemical measurements were performed using an Autolab PGSTAT 302 potentiostat from Eco Chemie (Utrecht, The Netherland). The experiments were carried out using a three-electrode system with a surface-confined polymer film on ITO as a working electrode, a Ag/AgNO_3_ non-aqueous reference electrode, and a Pt gauze counter electrode. The reference electrode was checked against the ferrocene standard every time before and after the experiments were performed, and the measured potentials were reported against the Fc/Fc^+^ redox potential value. All the electrochemical experiments were carried out in 0.1 M Bu_4_NPF_6_ solution in CH_2_Cl_2_ as the supporting electrolyte. Spin-cast films of the polymers **SP1** and **SP2** were prepared on quartz slides via spin-casting at 1000 rpm using 1 mg mL^−1^ solutions of polymers **SP1** and **SP2** in CHCl_3_.

### 3.2. Synthetic Procedures

**Triethoxy(4-iodophenyl)silane (1)** was synthesized following a modified literature procedure [49]. A solution of *i*-PrMgCl (0.7 mL of 1.85 M solution in THF; 6.67 mmol) was added dropwise to a solution of 2.0 g (6.06 mmol) of 1,4-diiodobenzene in 25 mL of anhydrous THF at −40 °C under a N_2_ atmosphere. The resulting mixture was stirred for 6 h to give a solution of (4-iodophenyl)magnesium chloride. Then, Si(OEt)_4_ (2 mL, 1.87 g, 18.2 mmol, *d* = 0.933 g cm^−3^) was added dropwise to the solution of (4-iodophenyl)magnesium chloride. The resulting solution was stirred at −40 °C for 1 h and then allowed to warm to room temperature overnight. The reaction mixture was concentrated in vacuo and purified by Kugelrohr distillation (0.01 mmHg, oven temperature 70 °C) to give 0.58 g (28%) of **1** as a clear colorless liquid: ^1^H NMR (CDCl_3_, 400 MHz) *δ* 7.73 (d, *J* = 8.0 Hz, 2H), 7.39 (d, *J* = 8.0 Hz, 2H), 3.85 (q, *J* = 6.8 Hz, 6H), 1.23 (t, *J* = 6.8 Hz, 9H).

**1,4-Diiodo-2,5-dimethoxybenzene (5)** was prepared as described in the literature [50].

**1,4-Dimethoxy-2,5-bis(trimethylsilylethynyl)benzene (S1).** A mixture of 1.51 g (15.39 mmol) of (trimethylsilyl)acetylene, 2.0 g (5.13 mmol) of **5**, 116 mg (0.1 mmol) of Pd(PPh_3_)_4,_ and 29 mg (0.15 mmol) of CuI in 150 mL of toluene–*i*-Pr_2_NH (7:3) solvent, was stirred at 70 °C in a sealed air-free flask for 48 h. After cooling down to room temperature, the mixture was concentrated in vacuo, and the crude product was purified via column chromatography on silica gel eluted with CH_2_Cl_2_–hexane (1:3) to afford 1.36 g (90%) of **S2** as a colorless solid, *R*_f_ 0.5, mp. 168–171 °C: ^1^H NMR (CDCl_3_, 400 MHz) *δ* 6.91 (s, 2H), 3.83 (s, 6H), 0.27 (s, 18H).

**1,4-Dimethoxy-2,5-diethynylbenzene (2).** A solution of 1.3 g (3.93 mmol) of **S1** in 5 mL of CH_2_Cl_2_ was added dropwise into a mixture of 5.44 g (39.3 mmol) of K_2_CO_3_ in 80 mL of MeOH—CH_2_Cl_2_ (1:1). The resulting mixture was stirred at room temperature for 1 h. The resulting solution was filtered, and the filtrate was collected and then diluted in 100 mL by adding CH_2_Cl_2_, washed with water three times, and then extracted with 100 mL of CH_2_Cl_2_ three times. The combined organic fraction was washed with brine, dried over Na_2_SO_4_, and concentrated in vacuo. The crude product was purified by column chromatography on silica gel eluted with CH_2_Cl_2_–hexane (1:3) to afford 0.70 g (95%) of **2** as a colorless solid, *R*_f_ 0.35, mp. 158–161 °C: ^1^H NMR (CDCl_3_, 400 MHz) *δ* 6.96 (s, 2H), 3.86 (s, 6H), 3.40 (s, 2H).

**1,4-Diiodo-2,5-bis(trifluoromethyl)benzene (4)** was synthesized following a modified literature procedure [51]. 1,4-Bis(trifluoromethyl)benzene (3.0 g, 14.01 mmol) was slowly added to a solution of 4.50 g (21.02 mmol) of NaIO_4_ and 6.98 g (42.03 mmol) of KI in 100 mL of H_2_SO_4_ at room temperature. The resulting mixture was stirred at 90 °C for 16 h. After cooling to room temperature, the reaction mixture was poured into ice, filtered, and washed with water. The solid was collected, washed with aqueous Na_2_S_2_O_3_, and extracted with CH_2_Cl_2_. The resulting organic fraction was washed with brine, dried over Na_2_SO_4_, concentrated in vacuo, and recrystallized from hexane to afford 3.24 g (50%) of **4** as a colorless solid, mp. 117–120 °C: ^1^H NMR (CDCl_3_, 400 MHz) *δ* 8.20 (s, 2H).

**1,4-Bis(trifluoromethyl)-2,5-bis(trimethylsilylethynyl)benzene (S2)** was prepared utilizing the same procedure as described above for **S1**. Compound **S2** (1.7 g, 97%) was obtained as a colorless solid: ^1^H NMR (CDCl_3_, 400 MHz) *δ* 7.83 (s, 2H), 0.27 (s, 18H).

**1,4-Bis(trifluoromethyl)-2,5-diethynylbenzene (6)**. A solution of 1.7 g (4.18 mmol) of **S2** in 5 mL of THF was added into a mixture of 5.78 g (41.82 mmol) of K_2_CO_3_ in 80 mL of MeOH—THF (1:1). The resulting mixture was stirred at room temperature for 1 h. The mixture was filtered, and the filtrate was collected and then diluted with 100 mL of CH_2_Cl_2_, washed with water three times, and then extracted with 100 mL of CH_2_Cl_2_ three times. The combined organic fraction was washed with brine, dried over Na_2_SO_4_, and concentrated in vacuo. The crude product was purified via column chromatography on silica gel eluted with CH_2_Cl_2_–hexane (1:3) to afford 0.99 g (90%) of **6** as a colorless solid, *R*_f_ 0.5, mp. 88–91 °C: ^1^H NMR (CDCl_3_, 400 MHz) *δ* 7.92 (s, 2H), 3.56 (s, 2H).

**4,7-Diiodobenzo[*c*][1,2,5]thiadiazole (3)** was prepared following the literature procedure [52].

**1,4-Bis(tetradecyloxy)-2,5-diethynylbenzene** (**S3**) was prepared as described in the literature [53].

**Soluble reference polymer SP1**. A mixture of 20 mg (0.036 mmol) of bis-acetylene **S3**, 14 mg (0.036 mmol) of 4,7-diiodobenzo[*c*][1,2,5]thiadiazole **3**, 0.8 mg (2 mol%) of Pd(PPh_3_)_4_, and 0.2 mg (2 mol%) of CuI in 5 mL of toluene–*i*-Pr_2_NH (7:3), was stirred at 70 °C in a sealed air-free flask for 72 h. After cooling down to room temperature, the mixture was poured into 50 mL of acetone, and the solid precipitate was separated via centrifugation, and dried in vacuo to afford 20 mg (80%) of **SP1** as a red solid, *M*_n_ 22.3 kDa, *Ð* 3.1 (GPC, vs. polystyrene): ^1^H NMR (CD_2_Cl_2_, 400 MHz) *δ* 7.82 (s, 2H), 7.19 (s, 2H), 4.17–4.05 (m, 4H), 1.97–1.87 (m, 4H), 1.36–1.17 (m, 44H), 0.94–0.83 (m, 6H).

**Soluble reference polymer SP2**, a mixture of 35 mg (0.064 mmol) of bis-acetylene **S3**, 30 mg (0.064 mmol) of diiodo monomer **4**, 1.4 mg (2 mol %) of Pd(PPh_3_)_4_ and 0.24 mg (2 mol %) of CuI in 10 mL of toluene–*i*-Pr_2_NH (7:3), was stirred at 70 °C in a sealed air-free flask for 72 h. After cooling down to room temperature, the mixture was poured into 50 mL of acetone, and the solid precipitate was separated via centrifugation and dried in vacuo to afford 30 mg (63%) of **SP2** as a yellow solid, *M*_n_ 5.9 kDa, *Ð* 1.8 (GPC, vs. polystyrene): ^1^H NMR (CDCl_3_, 400 MHz) *δ* 8.03–7.86 (m, 2H), 7.02 (s, 2H), 4.15–3.89 (m, 4H), 1.98–1.77 (m, 4H), 1.47–1.11 (m, 44H), 0.99–0.78 (m, 6H).

### 3.3. Surface-Confined Polymerization

#### 3.3.1. Activation of Substrates

**Activation of ITO-glass substrates.** Rectangular ITO-covered glass slides (approx. 1.1 × 2.5 cm) were ultrasonicated in CH_2_Cl_2_ for 20 min, followed by rinsing with acetone and deionized water. The pre-cleaned slides were subjected to an RCA-type cleaning procedure by keeping it in the mixture of water—30% H_2_O_2_—and 30% aqueous NH_3_ (5:1:1) at 70 °C for 1 h. The substrates were then rinsed with copious amounts of deionized water and dried in a N_2_ flow at room temperature overnight, and then activated using O_2_ plasma for 10 min.

**Activation of quartz substrates.** Rectangular quartz slides (approx. 1.1 × 2.5 cm) were ultrasonicated sequentially for 10 min in CHCl_3_, acetone, methanol, and deionized water. The pre-cleaned slides were placed into a Piranha solution (a mixture of conc. H_2_SO_4_ and 30% H_2_O_2_ (7:3)) and ultrasonicated for 30 min. After rinsing with a copious amount of deionized water, the substrates were dried in N_2_ flow at room temperature overnight, and then activated using O_2_ plasma for 10 min. ***NOTE:*** *extreme care must be taken when dealing with Piranha solutions as they can detonate when in contact with organic compounds!*

#### 3.3.2. Preparation of Thin Films by Surface-Confined Stepwise Sonogashira Polymerization

**Preparation of surface-immobilized monolayer of precursor 1.** Activated quartz or ITO substrates were immersed into a 10 mM solution of **1** in toluene and kept at 70 °C for 3 days. To obtain optimal results, immobilization was performed inside a glovebox. After deposition, the substrates were ultrasonicated sequentially for 10 min each in CHCl_3_, acetone, and deionized water, followed by drying under N_2_ flow overnight.

**Surface-immobilized thin films of polymer P1.** For the optimal results, this procedure was performed inside a glovebox with a nitrogen atmosphere. The substrates modified with a monolayer of initiator **1** were immersed into a 10 mM solution of the bis-acetylene monomer **2** in 7 mL of toluene–*i*-Pr_2_NH (7:3) containing 8 mg (0.007 mmol) of Pd(PPh_3_)_4_ and 1.3 mg (0.007 mmol) of CuI at 40 °C and kept there for 1 h upon the gentle stirring of the reacting solution (step A). After completing the coupling step, the substrates were rinsed twice with a copious amount of toluene, followed by ultrasonication in toluene for 3 min. Then, the substrates were placed into a 10 mM solution of the diiodo monomer **3** in 7 mL of toluene–*i*-Pr_2_NH (7:3) containing 8 mg (0.007 mmol) of Pd(PPh_3_)_4_ and 1.3 mg (0.007 mmol) of CuI at 40 °C and kept there for 1 h upon gentle stirring, followed by rinsing and ultrasonication in toluene (step B). This sequence of steps A and B was repeated eight more times to yield surface-immobilized thin films of polymer **P1**.

**Surface-immobilized thin films of polymers P2, P3, and P4.** These films were prepared following the procedure described above for the polymer **P1**. The following monomers were used: for **P2**, bis-acetylene **2** and diiodo monomer **4**; for **P3,** bis-acetylene **2** and diiodo monomer **5**; for **P4**, bis-acetylene **6** and diiodo monomer **4**.

**Surface-confined polymerization on silica microspheres.** Dried silica microspheres of 293 nm diameter (obtained from Bangs Laboratories, Inc., Fishers, IN, USA) in the amount of 500 mg were added to 15 mL of 10 mM solution of **1** in toluene, and the resulting suspension was stirred at 80 °C for 24 h. The microspheres were separated by centrifugation, and redispersed in toluene, ultrasonicated for 10 min, and again separated from the solvent by centrifugation. This washing procedure was repeated 2 more times to ensure complete removal of the free iodo precursor **1**. The surface-modified particles were added to a 7 mM solution of the bis-acetylene monomer **2** in 7 mL of toluene–*i*-Pr_2_NH (7:3) containing 6 mg (0.005 mmol, 10 mol%) of Pd(PPh_3_)_4_ and 1 mg (0.005 mmol, 10 mol%) of CuI, and the resulting mixture was vigorously stirred at 40 °C for 1 h (step A). The microspheres were separated by centrifugation, redispersed in dry THF and ultrasonicated for 5 min, followed by separation by centrifugation. This washing procedure was repeated 2 more times. The resulting microspheres were added to a 7 mM solution of the bis-iodo monomer **5** in 7 mL of toluene–*i*-Pr_2_NH (7:3) containing the same amounts of Pd(PPh_3_)_4_ and CuI as in step A, and the resulting mixture was vigorously stirred at 40 °C for 1 h (step B) and then subjected to the same washing procedure as described above for the step A. The combination of steps A and B was repeated seven more times. A portion of 100 mg of modified microspheres was withdrawn after 2, 4, and 6 (A + B) steps and dried in vacuo before further cleavage.

The cleavage procedure was as follows: a portion of 100 mg of the surface-modified particles was added to a mixture of 9 mL of DI water and 1 mL of HF (~50% aqueous solution), and the resulting mixture was stirred at room temperature for 6 h. Organic materials were extracted with CH_2_Cl_2_ and washed with water (3 times), dried over Na_2_SO_4_, and concentrated in vacuo. Only the oligomers **P3-2** (obtained after 2 (A + B) steps, approx. 0.5 mg) and **P3-4** (obtained after 4 (A + B) steps, approx. 0.3 mg) were obtained as yellow solids after this procedure (although **P3-4** was found barely soluble in organic solvents) and were analyzed by GPC. HF treatment of the microspheres obtained after 6 and 8 (A + B) steps resulted in insoluble solid materials that could not be separated and analyzed.

### 3.4. Preparation of Partially Deuterated Polymer **P1** Thin Films for Neutron Reflectometry Studies

**1,4-Dibromo-2,5-di(deuteriomethoxy)benzene (S4).** A mixture of 1.0 g (3.73 mmol) of 2,5-dibromohydroquinone, 1.35 g (9.33 mmol) of CD_3_I, and 2.06 g (14.92 mmol) of K_2_CO_3_ in 25 mL of DMF was stirred at 40 °C for 48 h. After cooling down to room temperature, the mixture was filtered and the filtrate was purified via column chromatography on silica gel (eluent CH_2_Cl_2_–hexane (3:1)) to yield 0.9 g (80%) of **S4** as a colorless solid, *R*_f_ 0.34, mp. 147–149 °C: ^1^H NMR (400 MHz, CDCl_3_) *δ* 7.10 (s, 2H).

**1,4-Di(deuteriomethoxy)-2,5-bis(trimethylsilylethynyl)benzene (S5).** A mixture of 0.71 g (7.22 mmol) of (trimethylsilyl)acetylene, 0.73 g (2.41 mmol) of **S4**, 139 mg (0.12 mmol) of Pd(PPh_3_)_4_, and 46 mg (0.24 mmol) of CuI in 100 mL of toluene–*i*-Pr_2_NH (7:3) was stirred at 70 °C in a sealed air-free flask for 48 h. After cooling down to room temperature, the mixture was concentrated in vacuo, and the crude product was purified by column chromatography on silica gel eluted with CH_2_Cl_2_–hexane (1:3) to afford 0.51 g (63%) of **S5** as a colorless solid, *R*_f_ 0.5: ^1^H NMR (CDCl_3_, 400 MHz) *δ* 6.90 (s, 2H), 0.27 (s, 18H).

**1,4-Di(deuteriomethoxy)-2,5-diethynylbenzene (2-D_6_).** A solution of 0.51 g (1.52 mmol) of **S7** in 10 mL of THF was added into a mixture of 204 mg (3.64 mmol) of KOH in 40 mL of MeOH—THF (1:1). The resulting mixture was stirred at room temperature for 1 h. The mixture was filtered, and the filtrate was collected and then diluted with 100 mL of CH_2_Cl_2_, washed with water three times, and then extracted three times with 100 mL of CH_2_Cl_2_. The combined organic fraction was washed with brine, dried over Na_2_SO_4_, and concentrated in vacuo. The crude product was purified by column chromatography on silica gel eluted with CH_2_Cl_2_–hexane (1:3) to afford 276 mg (95%) of **2-D_6_** as a colorless solid, *R*_f_ 0.35, mp. 162–165 °C: ^1^H NMR (CDCl_3_, 400 MHz) *δ* 6.98 (s, 2H), 3.40 (s, 2H).

**Surface-confined polymerization,** starting with a monolayer of **1** on quartz slides and using monomers **2-D_6_** and **3,** was carried out exactly as described above for the thin films of polymer **P1**.

### 3.5. X-Ray Photoelectron Spectroscopy (XPS)

The XPS experiments were carried out using a PHI VersaProbe II instrument (Physical Electronics, Chanhassen, MN, USA) equipped with a focused monochromatic Al K(alpha) source. A detailed description of XPS instrumentation and the experimental details on the estimation of the reaction conversion in the surface-confined Sonogashira cross-coupling are provided in Supporting Information.

### 3.6. Atomic Force Microscopy

Samples were characterized with a model 5500 atomic force microscope (AFM) equipped with Picoscan v5.3.3 software (Keysight Technologies, Englewood, CO, USA). The images were acquired using the contact mode in ambient conditions. Oxide-sharpened silicon nitride cantilevers with force constants ranging from 0.1 to 0.5 N m^−1^ were used for imaging (Veeco Probes, Santa Barbara, CA, USA). Digital images were processed with Gwyddion open-source software (version 2.9), which is supported by the Czech Metrology Institute (Jihlava, Czech Republic) [54].

### 3.7. Grazing Incidence X-Ray Scattering

Grazing incidence X-ray scattering measurements were performed at beamline 8-ID-E of the Advanced Photon Source at Argonne National Laboratory [55]. An X-ray wavelength of *λ* = 1.6868 Å was used. The area detector, a Pilatus 1M (Dectris, Baden, Switzerland) pixel array detector, was positioned 204 mm from the sample for the GIWAXS and 2165 mm from the sample for the GISAXS. In both geometries, the sample was measured under ambient conditions and the scattering was measured at two different detector heights for a range of incident angles, *θ* = 0.16° to 0.22°, and exposure times of 10 s (GIWAXS) or 15 s (GISAXS). Combining the corresponding images eliminated the rows of inactive pixels between the detector modules and verified that the samples were not damaged by the synchrotron beam. The acquired data (as two-dimensional images) were further treated and analyzed using *GIXSGUI* software package [56]. Fitting of the GISAXS data using a combined Porod plus Guinier model was performed with MATLAB (version R2015a).

### 3.8. Neutron Reflectometry

Neutron reflectivity measurements were performed at the Spallation Neutron Source Liquids Reflectometer (SNS-LR, Beamline 4B) at Oak Ridge National Laboratory. The reflectivity data were collected using a sequence of 3.25-Å-wide continuous wavelength bands (selected from 2.63 Å < *λ* < 16.63 Å) and incident angles (ranging over 0.60° < *θ* < 2.71°), where *λ* is the neutron wavelength and *θ* is the scattering angle. Using these settings, the momentum transfer, *q* = (4π sin *θ/λ*), was varied over a range of 0.008 Å^−1^ < *q* < 0.22 Å^−1^. Reflectivity curves were assembled by combining seven different wavelength and angle datasets together, maintaining a constant sample footprint and relative instrumental resolution of *δq*/*q* = 0.023 by varying the incident–beam apertures.

The reduced data consisted of absolute neutron reflectivity (*R*) vs. neutron momentum transfer *q*. *Layers* [57] and *Motofit* [58] software were used to fit the measured reflectivity curves, providing the reflectivity of a model scattering length density profile, which could be analyzed to determine the structure of the thin films. A one-layer model was used to model the depth profiles of the films. The scattering length density, thickness, and roughness of each layer was freely varied in the fitting procedure. The quality of fit was gauged by minimizing *χ*^2^ between the data and the model reflectivity curves.

### 3.9. Polarized Absorption Spectroscopy

The experiments were carried out using an Agilent Cary 5000 UV-Vis-NIR spectrophotometer that was equipped with a Harrick Glan Tompson polarizer (PTH-SMP) mounted before Brewster’s angle holder (BXH-S1G), and a depolarizer (DPS-R4V) installed after the holder in order to avoid the angular dependence of detector sensitivity to the plane-polarized light. The quartz slides with the surface-confined polymer **P3** (prepared in 9 (A + B) steps) were mounted on the holder, and could be rotated with respect to the vertical axis to the required values of the twisting angle *δ* (varied from 0 to 50° in 10° increments). The experimental setup is shown in Figure S10 in Supporting Information. For each value of angle *δ*, the dichroic ratio *D*_HV_ at 400 nm wavelength was determined as the ratio of the absorbance values of the polymer **P3** thin film sample obtained for horizontally (H) and vertically (V) polarized light and corrected for the absorbance of a corresponding isotropic sample (as an isotropic sample, a solution of oligomer **P3-2** in CH_2_Cl_2_ with optical absorbance similar to that of the **P3** film sample, placed in a quartz cuvette with 1 mm optical path, was used):

(2) $$D_{\mathrm{HV}}=\frac{A_{H}}{A_{V}}\left(\frac{A_{V}^{iso}}{A_{H}^{iso}}\right)$$

The following equation [37] displays the relationship between the dichroic ratio *D*_HV_ and the twisting angle *δ*:

(3) $$D_{\mathrm{HV}}=1+\frac{2-3\sin^{2}\psi }{\sin^{2}\psi }\sin^{2}\delta$$

where *ψ* is the tilting angle between the long molecular axis of the surface-confined conjugated polymer chains and the normal to the substrate surface. The plot of *D*_HV_ vs. sin^2^*δ* is shown in Figure S11 in Supporting Information. The tilting angle *ψ* of 58° was obtained from the slope of the linear dependence.

### 3.10. Fluorescent Sensing Studies with **P3** Thin Films

Quartz slides were carefully cut to a size that allowed them to fit tightly across the diagonal of a standard rectangular 1 cm quartz fluorescence cuvette. A thin film of polymer **P3** was prepared via a sequence of 9 (A + B) repeating coupling cycles as described above. The slide was placed in the cuvette, and it was successively filled with water to acquire an initial spectrum, and then with aqueous solutions of 2,4-DNT with increasing concentrations (prepared using sequential multiple dilution with water of a small aliquot of 2,4-DNT stock solution in ethanol). The fluorescence spectra were acquired at 45° angle with respect to incident light. A single slide was used to acquire a complete set of the spectra shown in Figure 7. For resetting/reusing experiments (Figure S14 in Supporting Information), after recording its fluorescent spectrum, a freshly prepared **P3** slide was first exposed to the 2,4-DNT aqueous solutions; the quenched slide was washed with a copious amount of ethanol. After recording its fluorescence spectrum, the slide was again exposed to the analyte solution, etc. The resetting and analyte exposure cycle could be repeated 3 times before the slide showed substantial degradation of the emissive properties.

## 4. Conclusions

We have developed a stepwise precision synthesis of poly(*p*-phenylene ethynylene) CP thin films using surface-confined Sonogashira cross-coupling. This method enables the effective tuning of the polymer electronic properties through the proper monomer choice, and delivers thin films with uniform parallel alignment of the polymer chains. This hard-to-achieve morphology of the thin films, together with the precise molecular structure of the monodisperse polymer chains, provides for the superior fluorescent chemosensing performance of these thin-film materials towards the detection of nitroaromatic analytes. Although further studies are needed to better understand the structural features of the surface-confined films, the efficient photoexcitation delocalization mechanism, and how they are related to the sensing performance, taken together with high mechanical and solvent stability originating from the covalent attachment of the polymer molecules to a substrate, these properties could make these materials advantageous for a range of other electronic and optoelectronic applications (especially those requiring charge transport in the direction normal to the film surface).

## Figures and Tables

**Table 1:** Electrochemical characterization of conjugated polymers prepared by surface-confined polymerization.

Polymer Film	*ϕ*_ox_, V, (vs. Fc/Fc^+^)/*E*_HOMO_, eV ^1^	*ϕ*_red_, V, (vs. Fc/Fc^+^)/*E*_LUMO_, eV ^2^	*E*_g_^ec^, eV ^3^	*E*_g_^opt^, eV ^4^
**P1**	0.54/–5.64	–0.86/–4.24	1.40	1.95
**P2**	0.96/–6.06	–1.24/–3.86	2.20	2.45
**P3**	0.53/–5.63	n.a. ^5^/–3.49 ^6^	n.a. ^5^	2.14
**P4**	n.a. ^7^/–6.59	–1.10/–4.00	n.a. ^5^	2.59

^1^ Calculated using the equation: *E*_HOMO_ = −(*ϕ*_ox_ + 5.1) (eV) [29]. ^2^ Calculated using the equation: *E*_LUMO_ = −(*ϕ*_red_ + 5.1) (eV) [29]. ^3^ Electrochemical energy gap. ^4^ Optical energy gap, estimated from the onset of the absorption band, as seen in Figure S8 in Supporting Information. ^5^ Not available since corresponding reduction wave was not observed. ^6^ Calculated using optical energy gap according to equation: *E*_g_^opt^ = *E*_LUMO_ − *E*_HOMO_. ^7^ Not available since corresponding oxidation wave was not observed.

## Data Availability

Data are contained within the article or Supplementary Materials.

## Supplementary Materials

The following supporting information can be downloaded at https://www.mdpi.com/article/10.3390/molecules29225466/s1, which provides the experimental details on the XPS experiments and the estimation of the reaction conversion in surface-confined Sonogashira cross-coupling; Table S1: fitting parameters for I 3*d*_5/2_ high-resolution XPS spectra in Figures S1 and S3; Figures S1–S3: high-resolution XPS spectra; Figure S4: GPC elution traces for oligomers **P3-2** and **P3-4**; Figure S5: UV/vis absorption spectrum of surface-confined polymer **P3** thin film prepared in 20 Sonogashira (A + B) coupling steps; Figure S6: cyclic voltammograms of surface-confined polymer thin films; Figure S7: evolution of the peak current vs. the number of Sonogashira (A + B) coupling steps in the synthesis of the polymer **P2** thin film; Figure S8: determination of the optical energy gap *E*_g_^opt^ from the onset of the UV/vis absorption spectra of the polymers **P1**–**P4**; Figure S9: AFM morphology of the thin film **P2**; Figure S10: AFM morphology of the quartz substrate used in the preparation of thin films; Figure S11: GISAXS and GIWAXS data for polymer **P2**; Figure S12: experimental setup for polarized absorption spectroscopy; Figure S13: dichroic ratio *D*_HV_ for a sample of surface-confined polymer **P3** as a function of the twisting angle *δ*; Figure S14: three 2,4-DNT fluorescent detection cycles upon exposing a polymer **P3** slide to fully quenching concentrations of the analyte followed by washing the exposed slide with ethanol; Figures S15–S27: ^1^H NMR spectra of the key compounds.
