# Supplementary material for: Precision Synthesis of Conjugated Polymer Films by Surface-Confined Stepwise Sonogashira Cross-Coupling
Source: Molecules. 2024 Nov 20;29(22):5466. doi: 10.3390/molecules29225466 (PMC11597661; doi:10.3390/molecules29225466)
Supplement: Supplementary file 1 [file molecules-29-05466-s001.zip › molecules-3277259-supplementary.pdf]

Supporting Information

Precision Synthesis of Conjugated Polymer Films by Surface-Confined Stepwise Sonogashira Cross-Coupling

Sang Gil Youm,[1] Mitchell T. Howell,[1] Chien-Hung Chiang,[2] Lu Lu,[2] Neepa M. K. Kuruppu Arachchige,[2] John F. Ankner,[3] Joseph Strzalka,[4] Yaroslav Losovyj,[5] Jayne C. Garno,[2] and Evgueni E. Nesterov,[1,2]*

[1]Department of Chemistry & Biochemistry, Northern Illinois University, DeKalb, Illinois 60115, USA

[2]Department of Chemistry, Louisiana State University, Baton Rouge, Louisiana 70803, USA

[3]Spallation Neutron Source, Oak Ridge National Laboratory, Oak Ridge, Tennessee 37831, USA

[4]X-ray Science Division, Argonne National Laboratory, Argonne, Illinois 60439, USA

[5]Department of Chemistry, Indiana University, Bloomington, Indiana 47401, USA

Corresponding Author

E-mail: een@niu.edu (E.E.N.).

I. Additional Experimental Details.

X-ray photoelectron spectroscopy (XPS). The XPS experiments were carried out using PHI *VersaProbe II* instrument equipped with a focused monochromatic Al K(alpha) source. Instrument base pressure was ca. 8×10^{-10} Torr. The X-ray power of 25 W at 15 kV was used for all experiments with 100 micron beam size at the X-ray incidence and take off angles of 45°. The instrument work function was calibrated to give a binding energy (BE) of 84.0 eV for Au $4f_{7/2}$ line for metallic gold and the spectrometer dispersion was adjusted to give a BE's of 284.8 eV, 932.7 eV and of 368.3 eV for the C 1*s* line of adventitious (aliphatic) carbon presented on the non-sputtered samples, Cu $2p_{3/2}$ and Ag $3d_{5/2}$ photoemission lines, respectively. The patented PHI dual charge neutralization system was used on all samples. The high resolution I 3*d* and C 1*s* spectra were taken with a minimum of 10-60 s scans using 0.1 eV steps and 46.95 eV and 11.75 eV pass energy, respectively. Signal above background measurement and Shirley background subtraction was made using *MultiPak* v9.0 PHI software. At the ultimate *Versa Probe II* instrumental resolution the temperature spread (at 14/86%) of the metallic silver Fermi edge was less than 120 meV. All XPS spectra were recorded using PHI software *SmartSoft –XPS* v2.0 and processed using PHI *MultiPack* v9.0 and/or *CasaXPS* v.2.3.14. The relative sensitivity factors from *MultiPack* library were used to determine atomic percentages. Peaks were fitted using GL line shapes a combination of Gaussians and Lorentzians. Wherever possible, conclusions were drawn from the number of resolved signals for a given element, so as to minimize reliance on absolute binding energies for the nonconductive molecular materials. A given sample was examined at 5-6 different spots on the mounted specimen to assure that consistent, reproducible results were obtained.

The high resolution I $3d_{5/2}$ XPS spectra have been deconvoluted into two components I_1 and I_2 which generated reasonably good fits (red curves in Figures S1 and S3). The fitting parameters as well as atomic ratios of the I_2 component to C 1*s* signal are presented in Table S1.

Table S1. Fitting parameters for I 3$d_{5/2}$ high-resolution XPS spectra in Figures S1 and S3.

Trace/ Sample	I 3$d_{5/2}$ components	Position, eV	Position separation, eV	FWHM, eV	% Gauss	% Area	Ratio I_2 3$d_{5/2}$/C 1s ×10^{-3}	Chi Squared
**Fig. S1**								
Upper spectra (30 min)	I_1 green	618.90	0.0	1.87	94	46.45	4.95	1.07
	I_2 blue	620.46	1.56	1.77	90	54.55		
Middle spectra (1 h)	I_1 green	619.08	0.0	1.75	99	83.33	2.57	1.54
	I_2 blue	620.5	1.42	1.75	88	16.67		
Bottom spectra (2 h)	I_1 green	619.11	0.00	1.45	90	88.5	0.838	1.79
	I_2 blue	620.71	1.6	1.5	90	11.5		
**Fig. S3**								
Bottom spectra	I_1 green	618.11	0.0	2.48	100	17.19	2.00	0.95
	I_2 blue	620.76	2.66	3.2	80	82.81		

Estimation of the reaction conversion in surface-confined Sonogashira coupling.

Step 1 A:

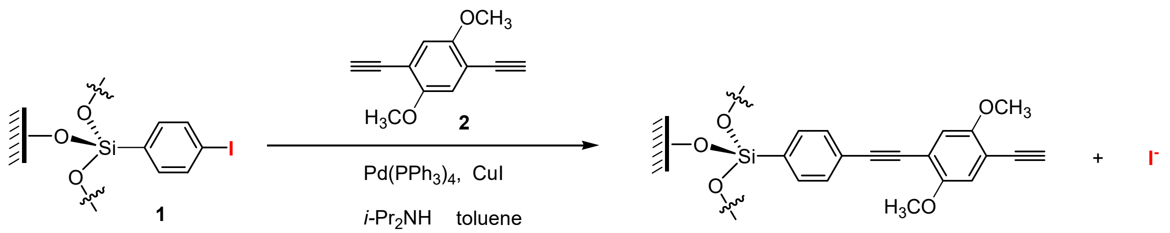

The quartz slides with a covalently immobilized monolayer of iodo-precursor **1** were placed in a solution containing bis-acetylene monomer **2** and catalytic amounts of $Pd(PPh_3)_4$ and CuI at 40 °C for various time intervals (ranging from 30 min to 2 h) to yield an alkyne-terminated monolayer-coupling product (step 1A). After the slides were thoroughly rinsed with toluene to remove excess reactants, catalysts, and reaction byproducts, the samples were analyzed by XPS (Figures S1 and S2). The iodine signals of **1** corresponding to I 3$d_{5/2}$ and I 3$d_{3/2}$ peaks were observed at approximately 621 and 632 eV, and their intensity could be compared with the intensity of the C 1s signal at 285 eV. Further analysis of the residual iodine signals revealed the presence of two kinds of iodine – a less electron-rich iodobenzene-type iodine from unreacted compound **1**, and a more electron-rich iodide anion resulting from Sonogashira coupling of **1**

(possibly trapped inside the film). Deconvolution of the individual signals was done on the I 3$d_{5/2}$ signal and allowed quantification of both contributors – a peak from **1** at 621 eV, and an iodide anion peak at 619 eV (Figure S1). The iodo-initiator compound **1** has 6 C atoms, and the Sonogashira coupling product has 18 C atoms. If the reaction conversion is x, then the observed atomic fraction of unreacted iodine from **1** in the monolayer is $(1 - x)$, whereas the atomic fraction of carbon is $6 \times (1 - x) + 18x = 6 + 12x$ (since carbon from both **1** and the coupling product will be quantified together). Therefore, the atomic ratio of iodine (from **1**) to carbon will be given as:

$$\frac{\mathrm{I}}{\mathrm{C}} = \frac{1 - x}{6 + 12x}$$

Using the atomic ratios obtained from XPS analysis for various reaction times and provided in the last column of Table S1 (30 min: 0.00495; 1 h: 0.00257; 2 h: 0.000838) and solving for x allowed estimating conversions at these time intervals: 30 min: 0.92; 1 h: 0.96; and 2 h: 0.99. Considering particular sensitivity of XPS method to probing outside surface of the film (and thus some misrepresentation of the residual I deeper inside the film at higher conversions), these estimates can be considered the minimal possible values, and the actual conversion values could be even higher.

Step 2 A:

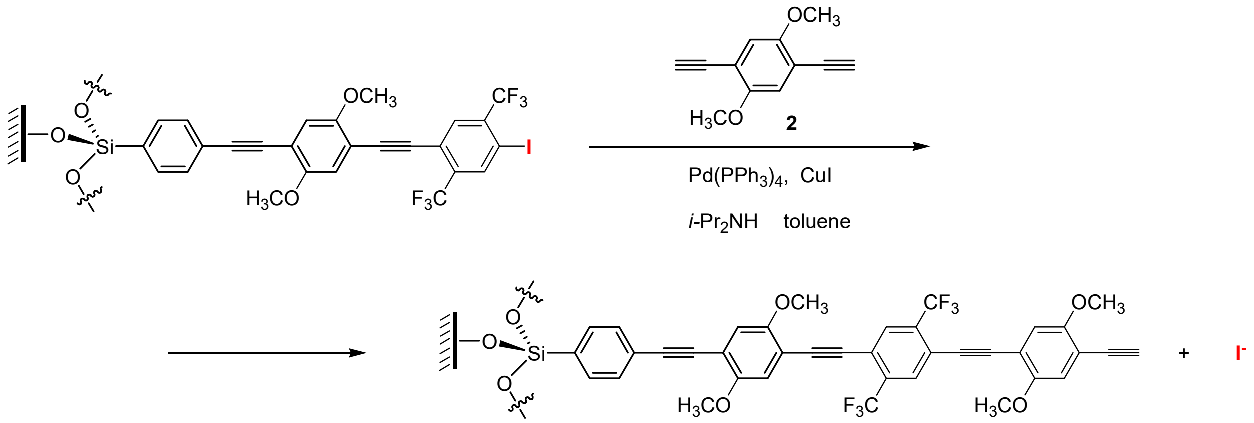

A similar analysis as for the step 1A yielded the following equation for the atomic ratio of iodine to carbon in the monolayer (using the experimental value from XPS analysis):

$$\frac{\mathrm{I}}{\mathrm{C}} = \frac{1 - x}{26 + 12x} = 0.002$$

Solving this equation for the reaction conversion x gave its value at 0.93. As in the case above, this should be considered the minimal possible value, and actual reaction conversion could be actually higher.

II. Additional Figures.

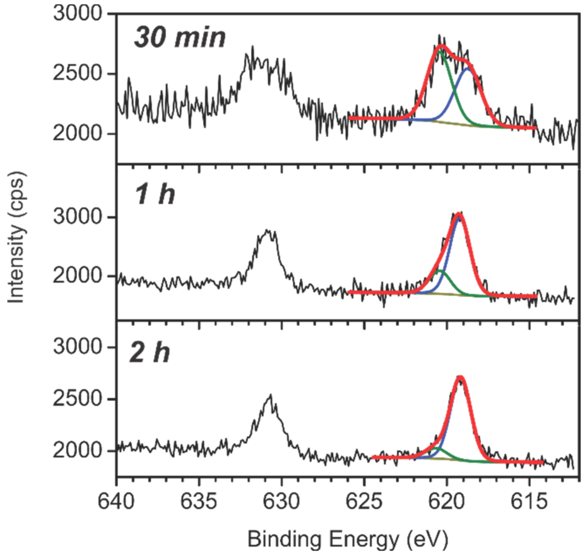

Figure S1. High-resolution I 3*d* XPS spectra of surface-immobilized monolayer of **1** after reaction with monomer **2** for the specified amounts of time. Deconvolution into two I $3d_{5/2}$ components is also shown. Shirley background (yellow traces) was applied to the spectra. Fitting parameters and deconvolution details are listed in Table S1.

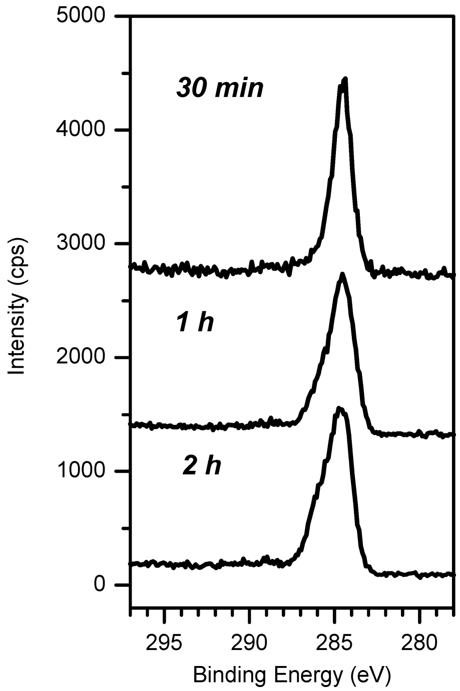

Figure S2. High-resolution C 1*s* XPS spectra of surface-immobilized monolayer of **1** after reaction with monomer **2** for the specified amounts of time.

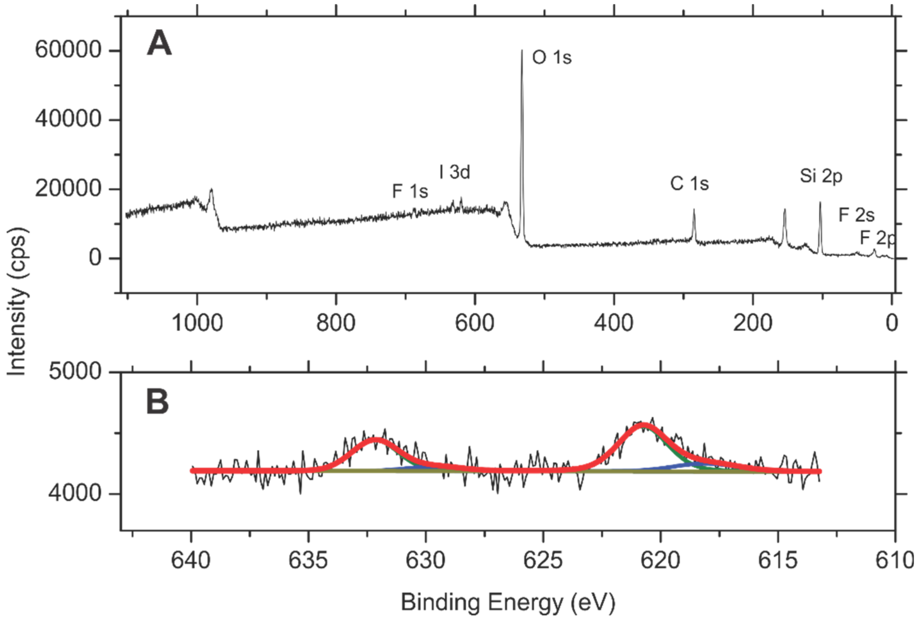

Figure S3. High-resolution XPS spectra of a surface-immobilized oligomer corresponding to polymer **P2** on quartz substrate after reaction cycle 2, step A. A) Survey spectrum. B) High-resolution I 3*d* XPS spectrum. Deconvolution into two I $3d_{5/2}$ and $3d_{3/2}$ components is also shown. Shirley background (yellow traces) was applied to the spectra. Fitting parameters and deconvolution details are listed in Table S1.

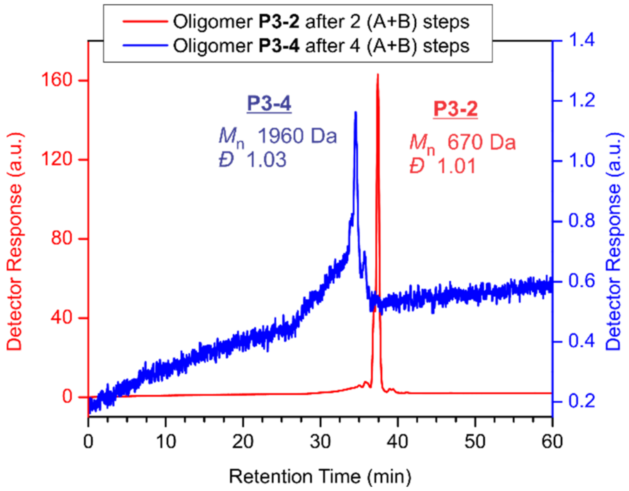

Figure S4. GPC elution traces for oligomers **P3-2** and **P3-4**. The detector response axes are color-matched with the corresponding elution traces.

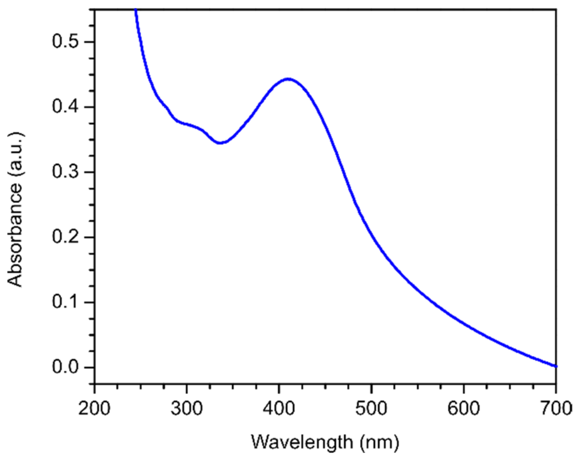

Figure S5. UV/vis absorption spectrum of surface-confined polymer **P3** thin film prepared in 20 Sonogashira (A+B) coupling steps.

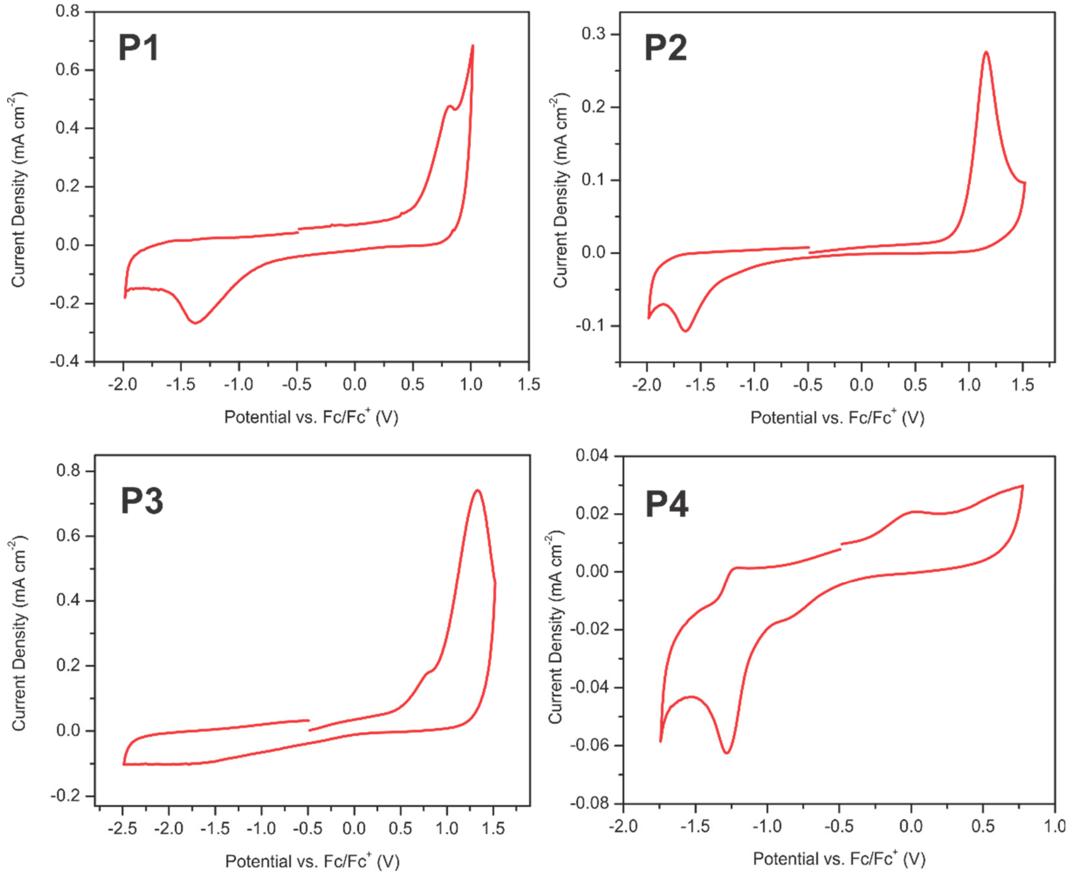

Figure S6. Cyclic voltammograms of surface-confined polymer thin films prepared on ITO. Data were acquired in 0.1 M Bu_4NPF_6 in CH_2Cl_2, with sweep rate 0.1 V s^{-1}.

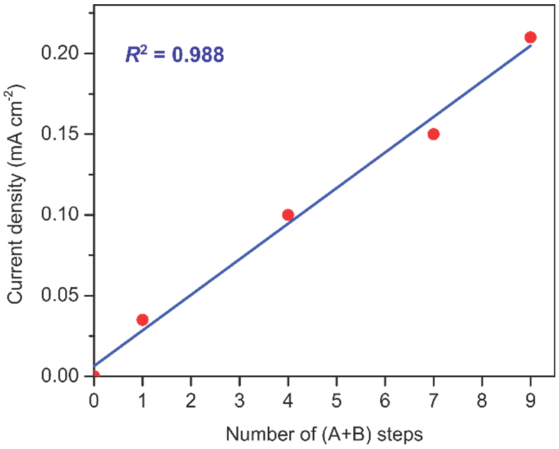

Figure S7. Evolution of peak current (corresponding to the oxidation peak at 1.05 V) vs. number of Sonogashira (A+B) coupling steps in synthesis of the polymer **P2** thin film. The films were prepared on ITO. Data were acquired in 0.1 M Bu_4NPF_6 in CH_2Cl_2, with sweep rate 0.1 V s^{-1}.

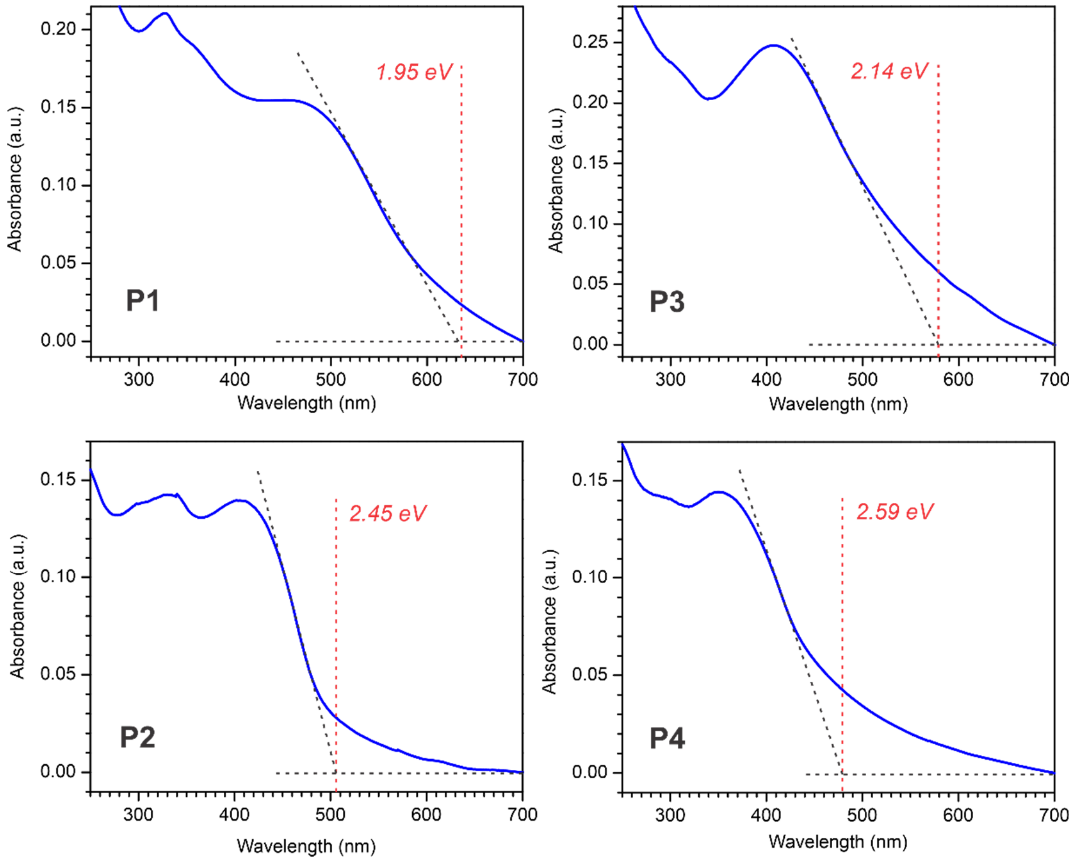

Figure S8. Determination of optical energy gap E_g^{opt} from the onset of UV/vis absorption spectra of the polymers **P1** – **P4**.

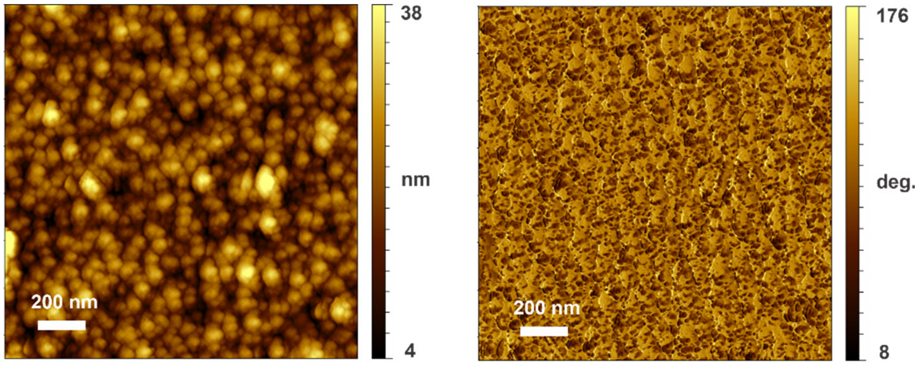

Figure S9. Morphology of the thin film **P2** as viewed with contact mode AFM – topography (left image) and a simultaneously acquired lateral force image (right image).

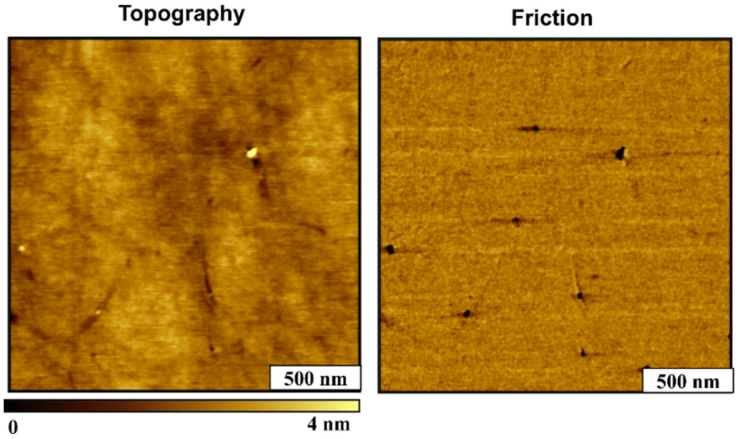

Figure S10. Morphology of the quartz substrate used in preparation of thin films as viewed with contact mode AFM – topography and a simultaneously acquired lateral force image.

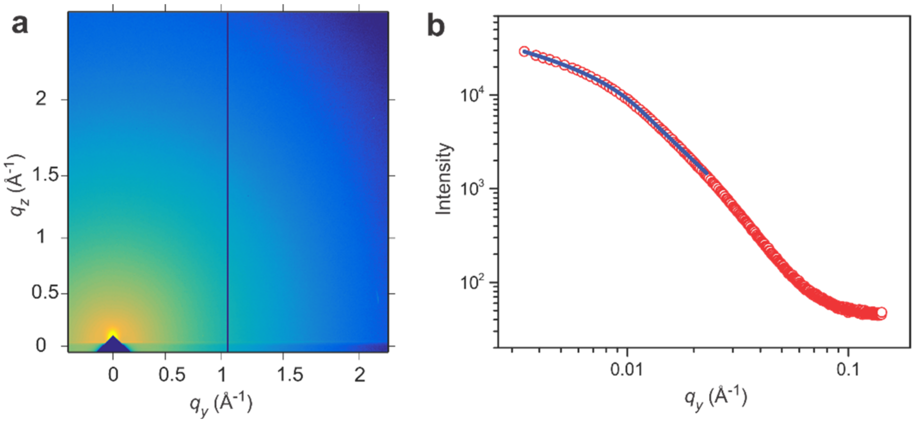

Figure S11. a) Two-dimensional GIWAXS image of a thin film of polymer **P2** on a quartz substrate. b) GISAXS horizontal line trace for the film in (a) (red circles) and fitting these data using a modified Guinier-Porod model (blue trace).

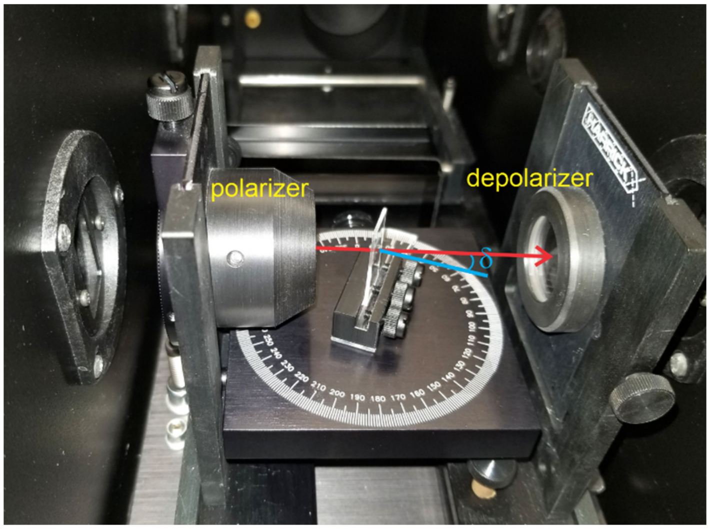

Figure S12. Experimental setup for polarized absorption spectroscopy mounted inside the sample compartment of Cary 5000 spectrometer. The twisting angle δ (the angle between the optical path direction and the normal to sample slide) was changed from 0 to 50° in 10° increments.

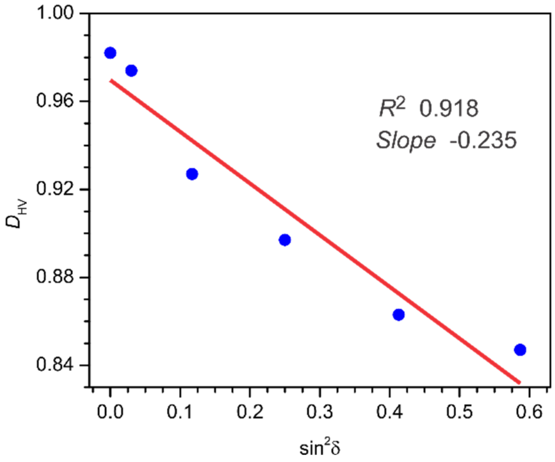

Figure S13. Dichroic ratio D_{HV} for a sample of surface-confined polymer **P3** as function of twisting angle δ. The absorbance data were obtained at 400 nm; the straight line is linear interpolation of the experimental data.

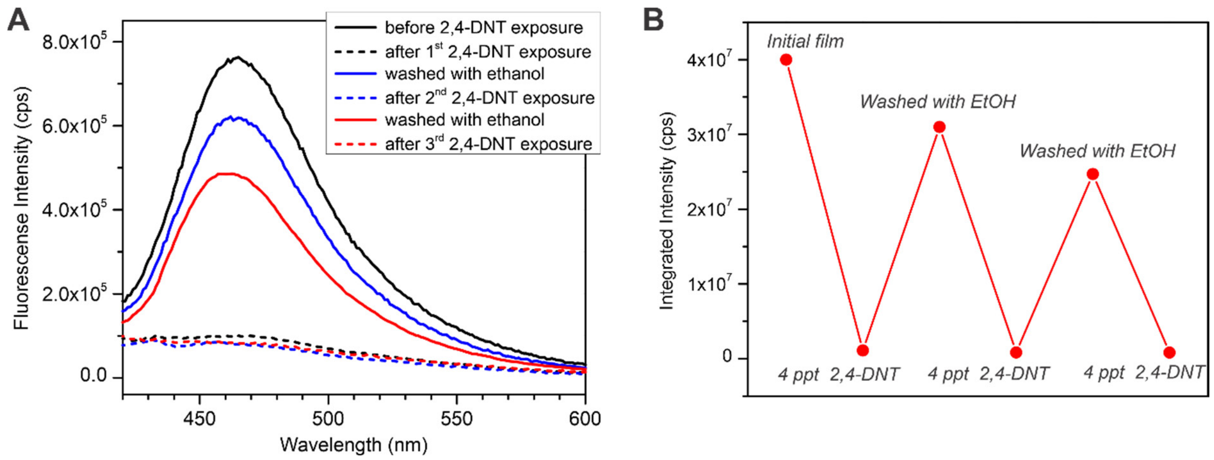

Figure S14. Three 2,4-DNT fluorescent detection cycles upon exposing a polymer **P3** slide to fully quenching concentrations of the analyte followed by washing the exposed slide with ethanol. A) Emission spectra. B) Change of total integrated intensity of the emission band.

^{1}H NMR spectra of the key compounds.

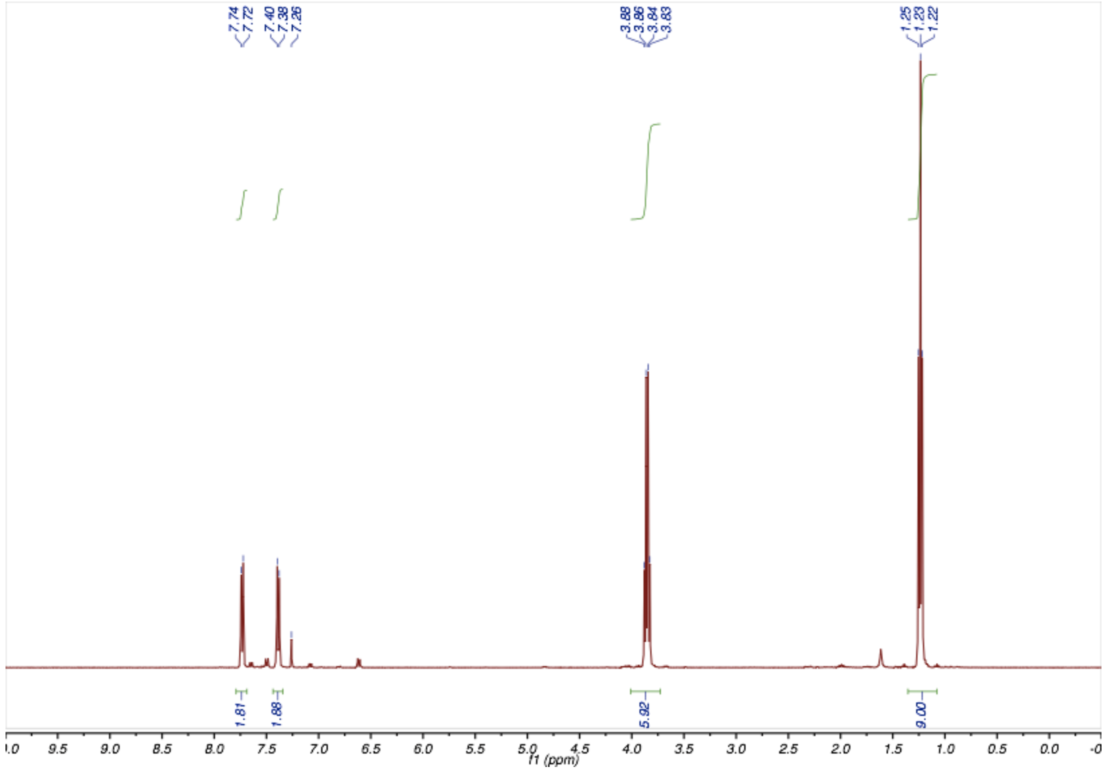

Figure S15. ^{1}H NMR spectrum of compound **1** ($CDCl_3$, 400 MHz).

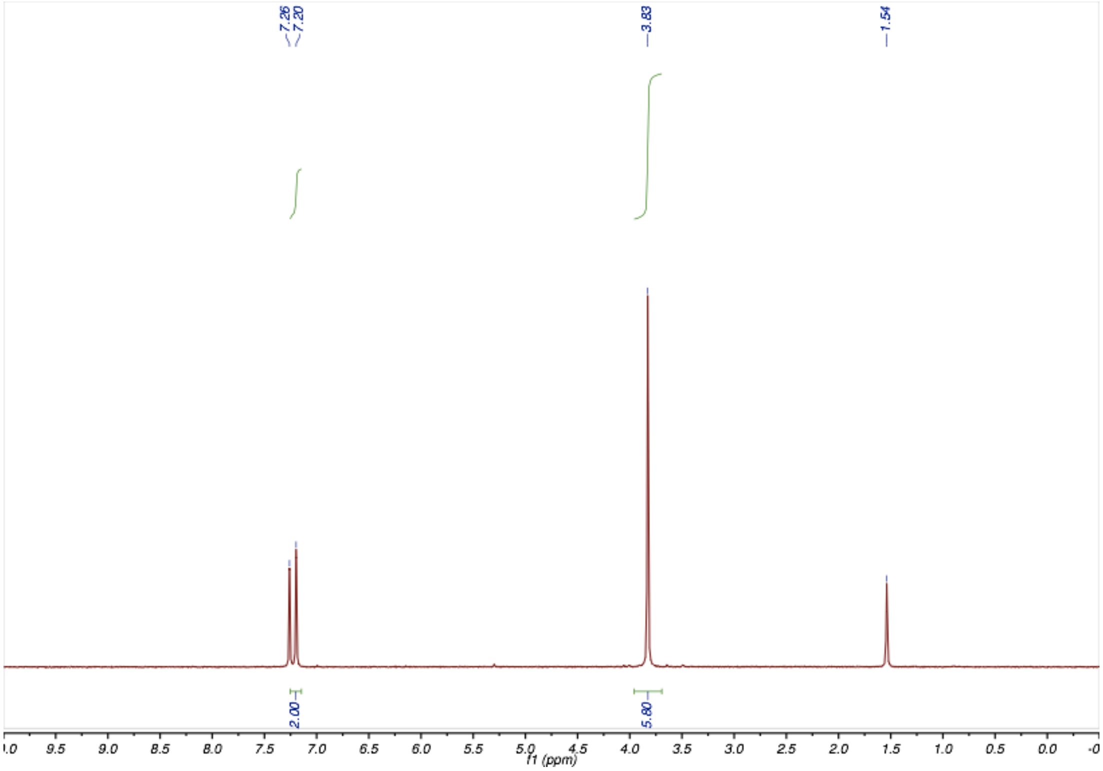

Figure S16. ^{1}H NMR spectrum of compound **5** ($CDCl_3$, 400 MHz).

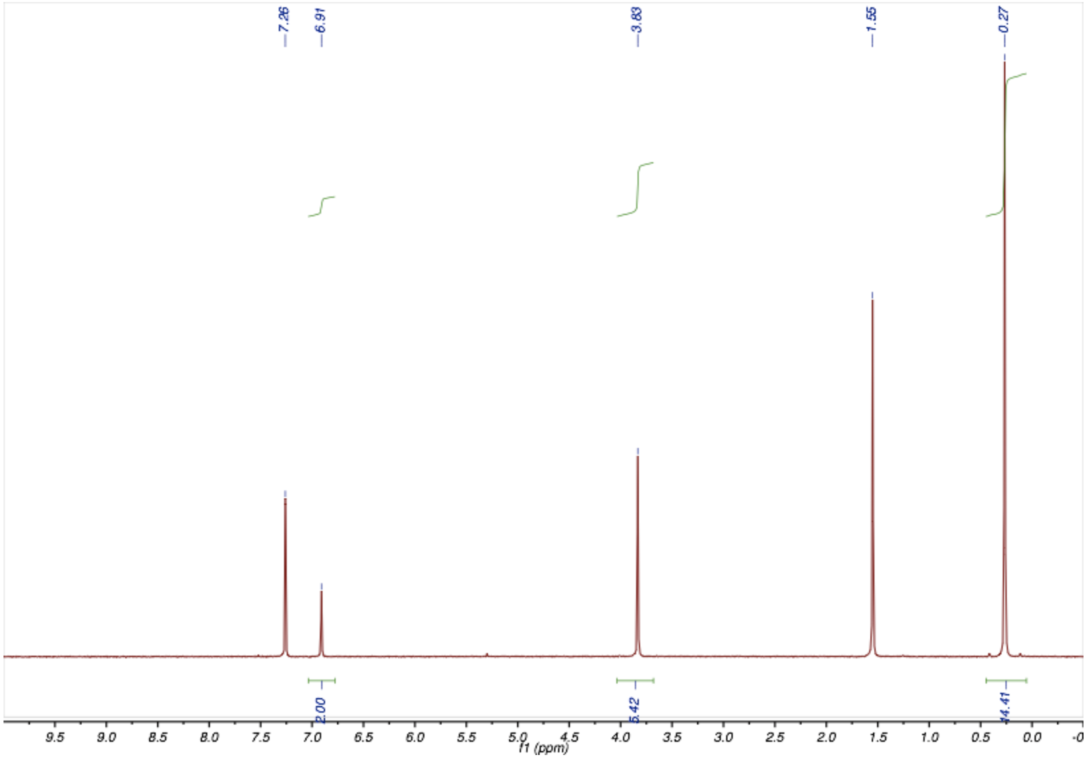

Figure S17. ^{1}H NMR spectrum of compound **S1** ($CDCl_3$, 400 MHz).

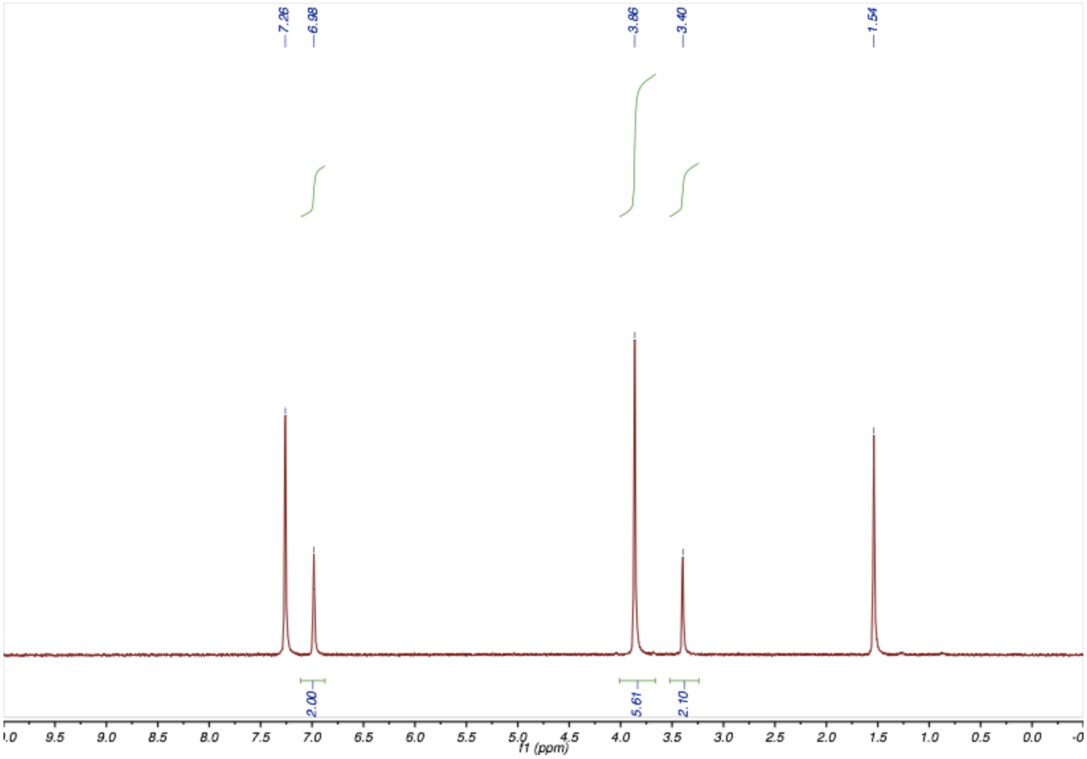

Figure S18. ^{1}H NMR spectrum of compound **2** ($CDCl_3$, 400 MHz).

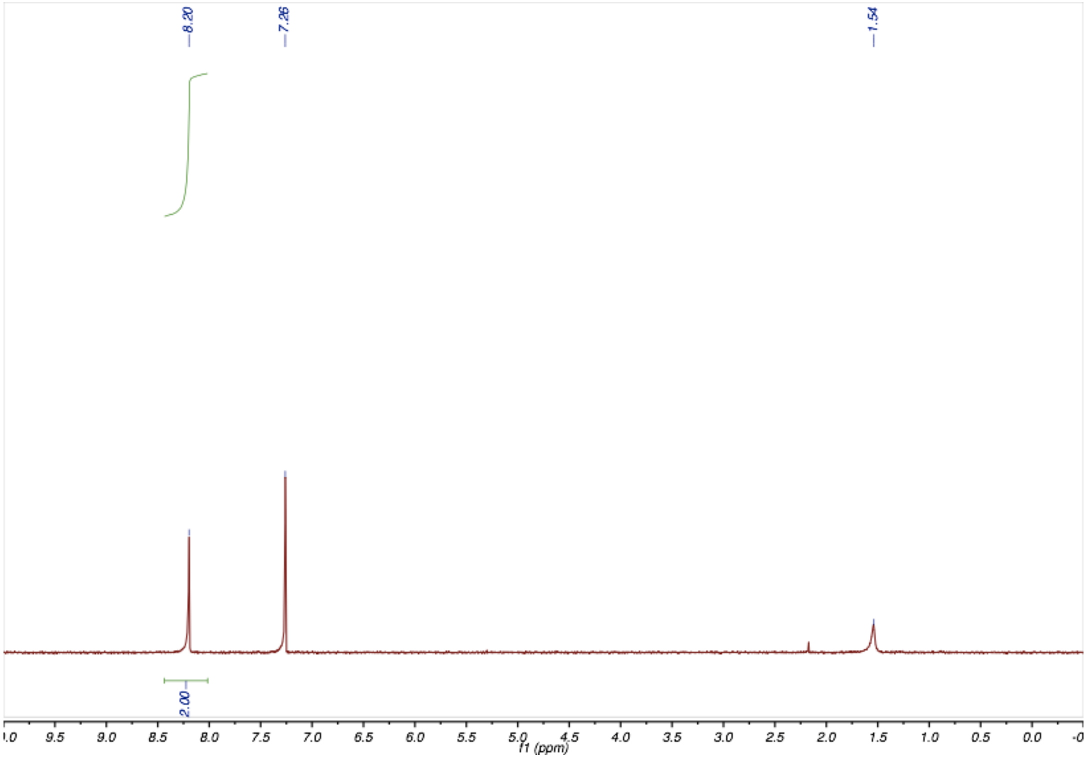

Figure S19. ^{1}H NMR spectrum of compound **4** ($CDCl_3$, 400 MHz).

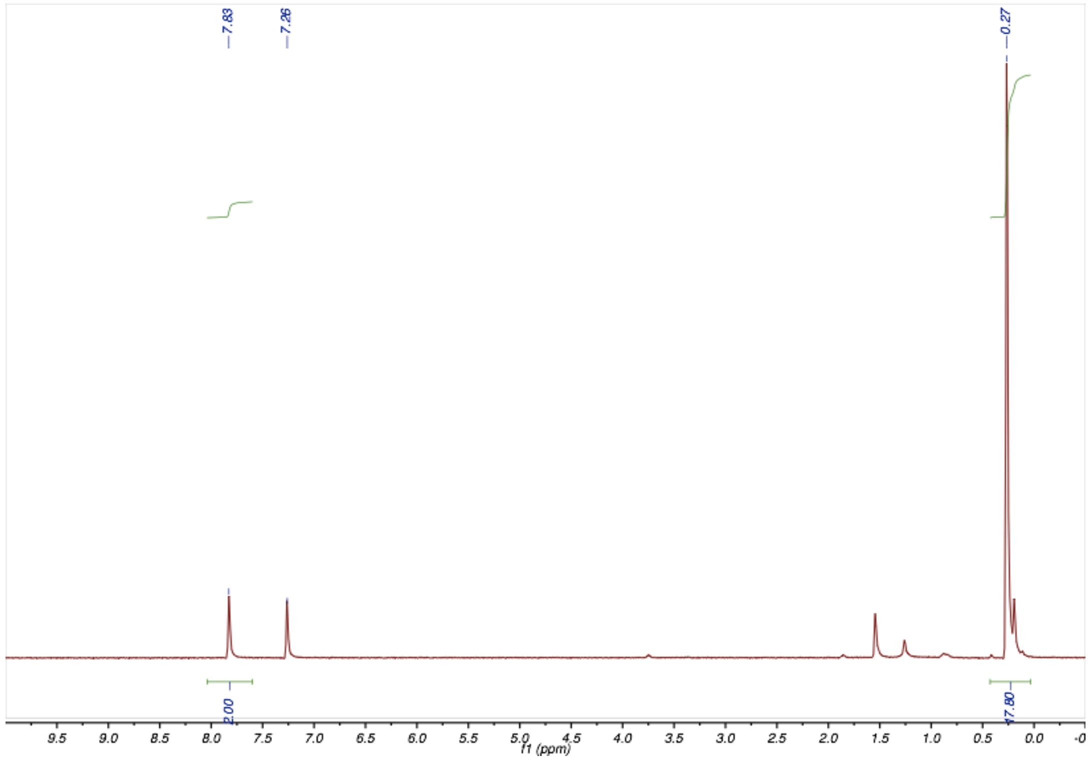

Figure S20. ^{1}H NMR spectrum of compound **S2** ($CDCl_3$, 400 MHz).

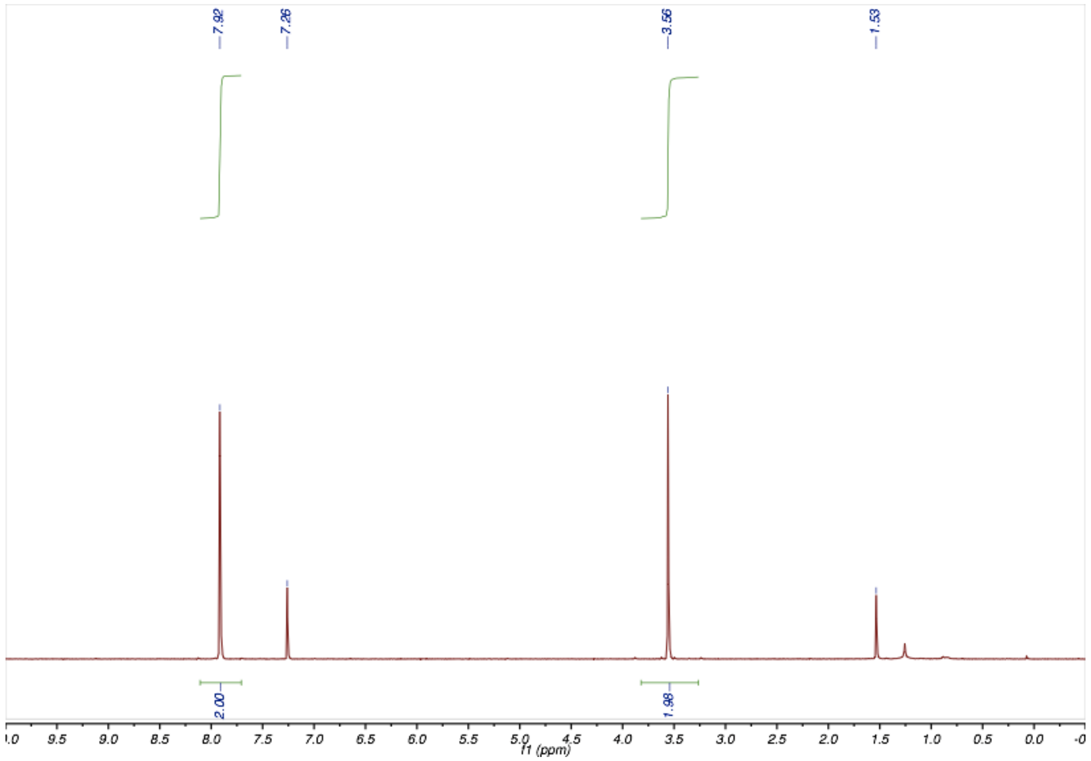

Figure S21. ^{1}H NMR spectrum of compound **6** ($CDCl_3$, 400 MHz).

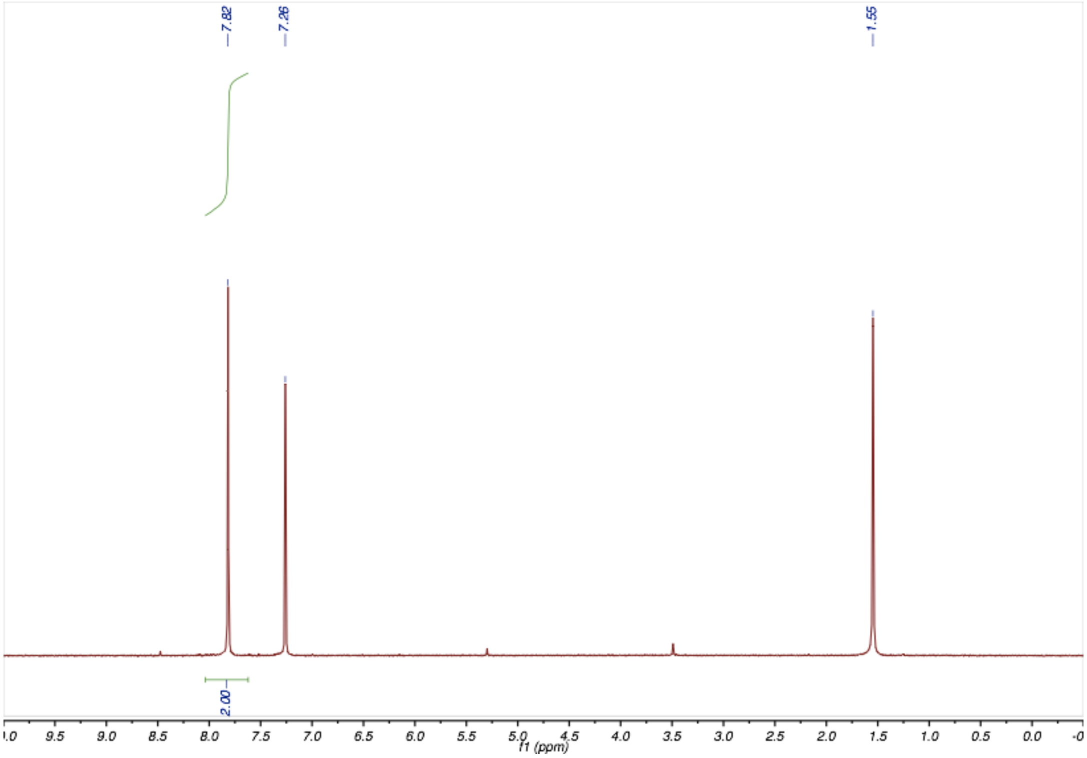

Figure S22. ^{1}H NMR spectrum of compound **3** ($CDCl_3$, 400 MHz).

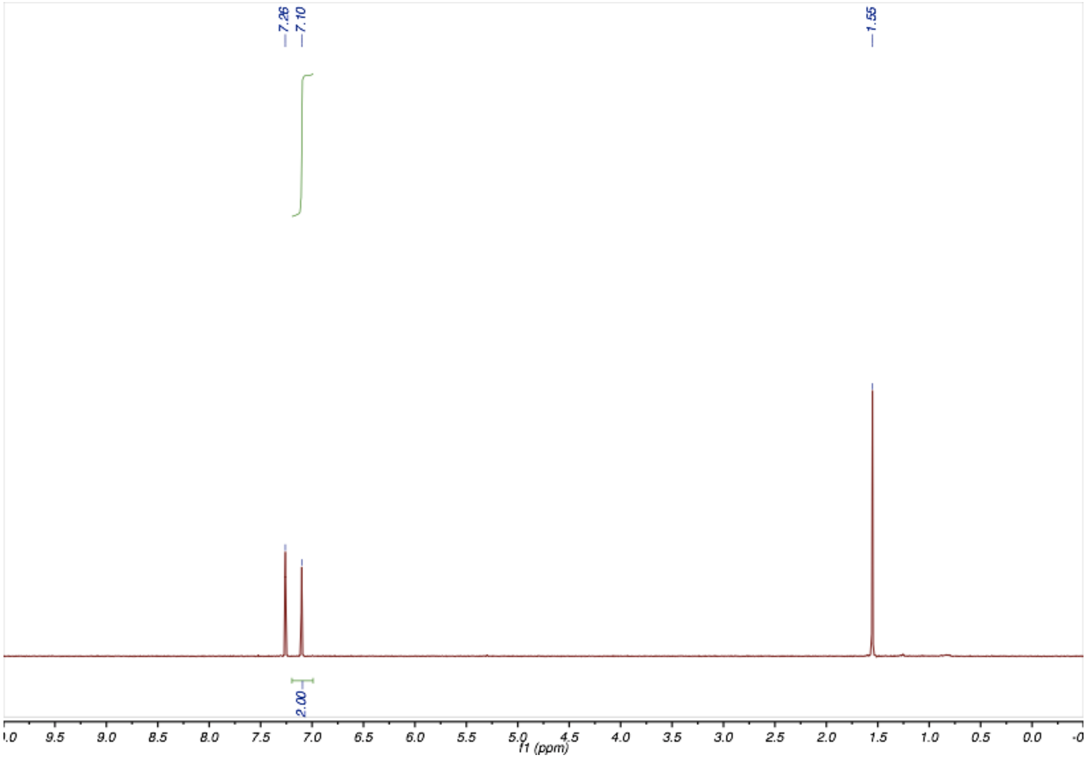

Figure S23. ^{1}H NMR spectrum of compound **S4** ($CDCl_3$, 400 MHz).

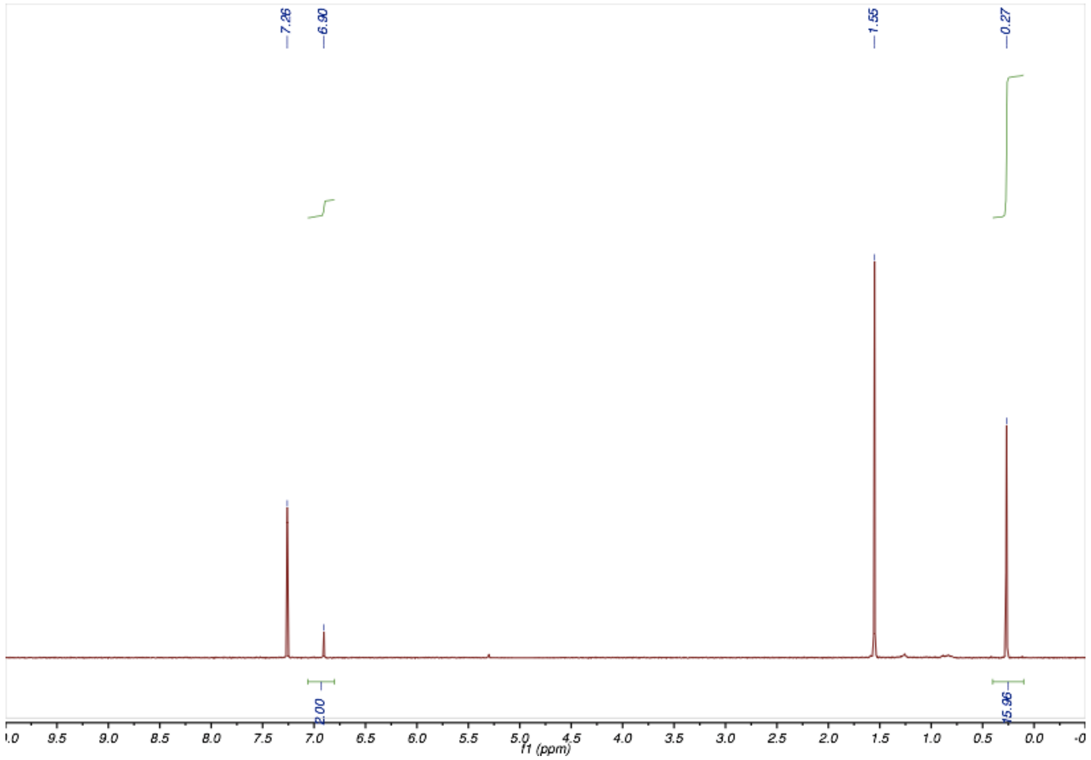

Figure S24. ^{1}H NMR spectrum of compound **S5** ($CDCl_3$, 400 MHz).

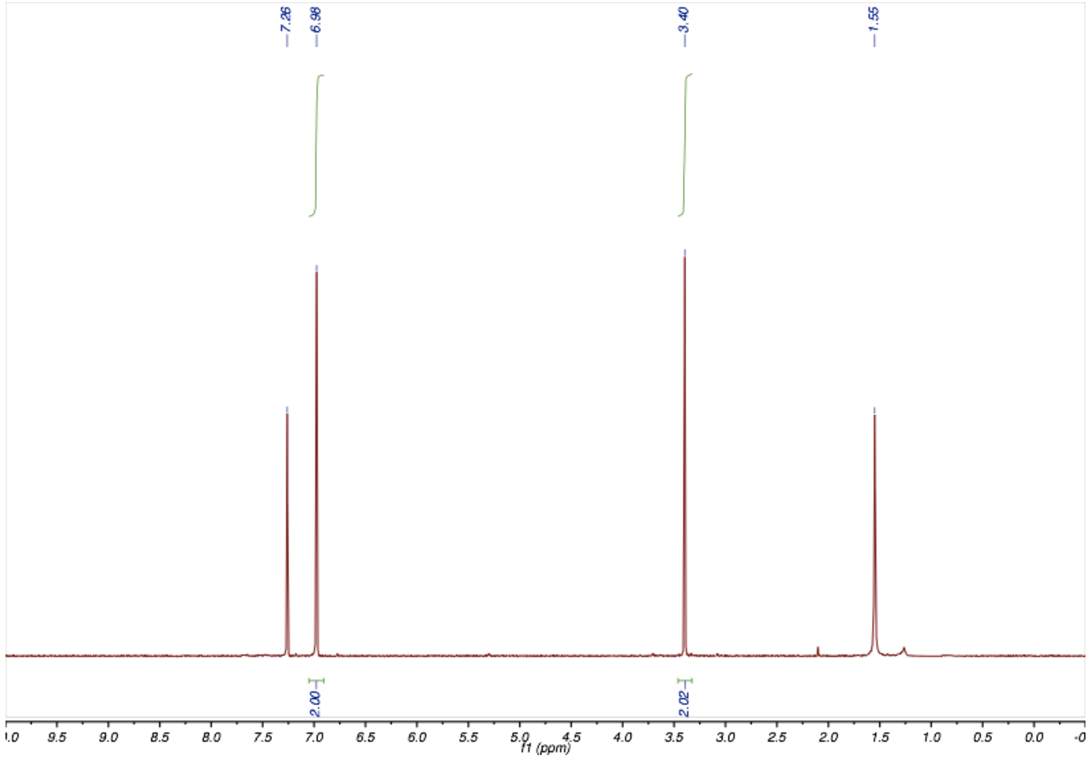

Figure S25. ^{1}H NMR spectrum of compound **2-D$_6$** ($CDCl_3$, 400 MHz).

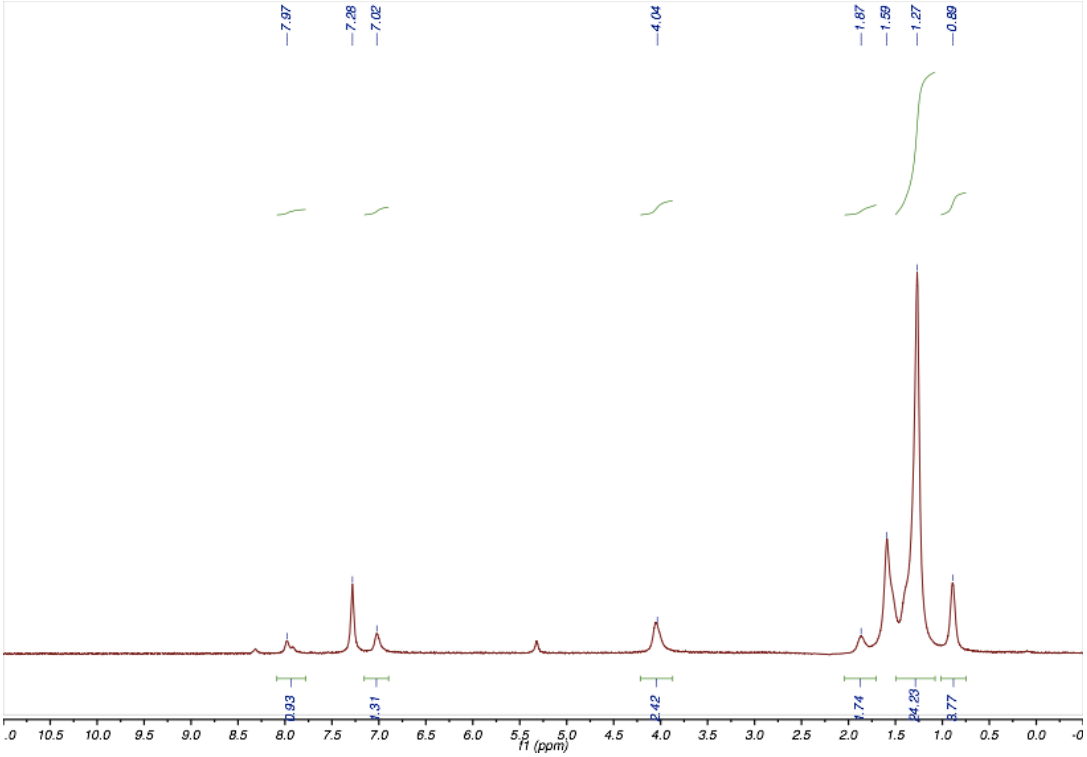

Figure S26. ^{1}H NMR spectrum of polymer **SP1** ($CDCl_3$, 400 MHz).

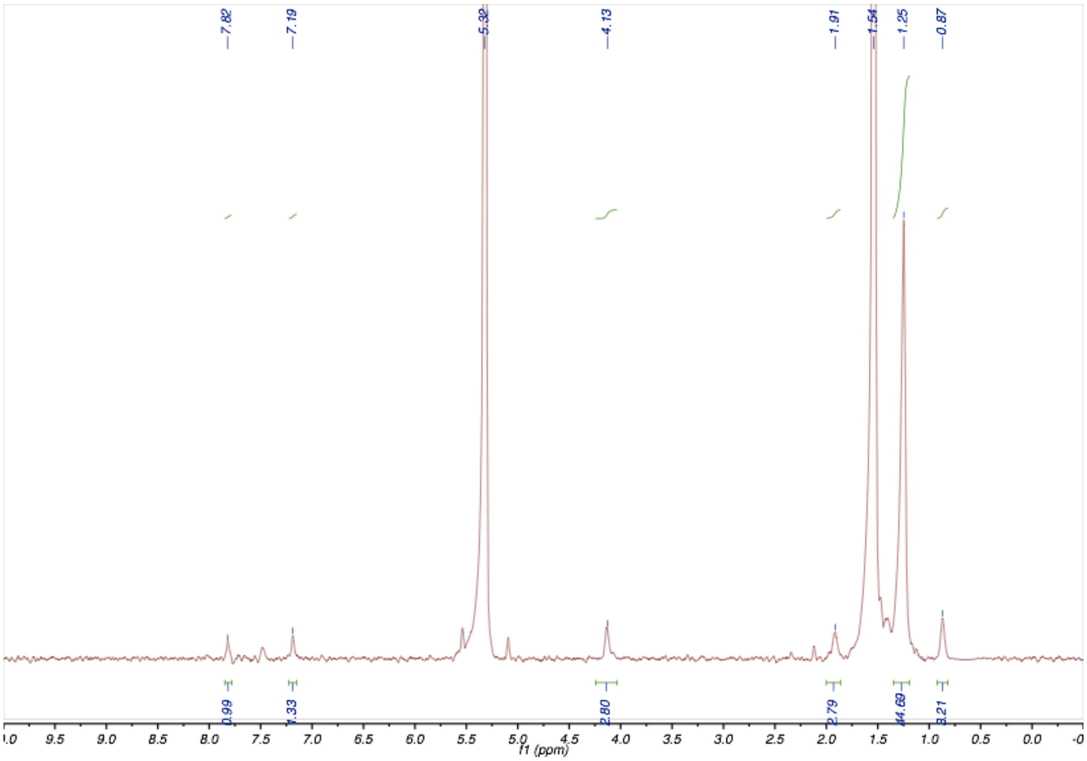

Figure S27. ^{1}H NMR spectrum of polymer **SP2** (CD_2Cl_2, 400 MHz).
